# Supplementary material for: Deciphering the unusual fluorescence in weakly coupled bis-nitro-pyrrolo[3,2-b]pyrroles
Source: Commun Chem. 2020 Dec 17;3:190. doi: 10.1038/s42004-020-00434-6 (PMC9814504; doi:10.1038/s42004-020-00434-6)
Supplement: Supplementary file 1 — Supplementary Information [file 42004_2020_434_MOESM1_ESM.docx]

Supplementary Information

**Deciphering the Unusual Fluorescence in Weakly Coupled**

**Bis-nitro-pyrrolo[3,2-*b*]pyrroles**

Yevgen M. Poronik,^1^ Glib V. Baryshnikov,^2^ Irena Deperasińska,^3^ Eli M. Espinoza,^4,#^ John A. Clark,^5^ Hans Ågren,^2,6^*** Daniel T. Gryko,^1^,* and Valentine I. Vullev^4,5,7,8^,**

^1^Institute of Organic Chemistry, Polish Academy of Sciences, Warsaw, Poland

^2^Department of Physics and Astronomy, Uppsala University, Uppsala, Sweden

^3^Institute of Physics, Polish Academy of Sciences, Warsaw, Poland

^4^Department of Chemistry, University of California, Riverside, U.S.A.

^5^Department of Bioengineering, University of California, Riverside, U.S.A.

^6^College of Chemistry and Chemical Engineering, Henan University, Kaifeng, Henan 475004, P.R. China

^7^Department of Biochemistry, University of California, Riverside, U.S.A.

^8^Materials Science and Engineering Program, University of California, Riverside, U.S.A.

^#^Present address: College of Bioengineering, University of California, Berkeley, CA 94720, U.S.A.

*Correspondence: dtgryko@icho.edu.pl

**Correspondence: vullev@ucr.edu

***Correspondence: hagren@kth.se

**Supplementary Methods**

1. **General chemical synthesis methods**

All chemicals were used as received unless otherwise noted. All reported ^1^H NMR spectra were collected using 500 and 600 MHz spectrometers. Chemical shifts (*δ* ppm) were determined with TMS as the internal reference; *J* values are given in Hz. The mass spectra were obtained via EI-MS and ESI-MS methods. All melting points for crystalline products were measured with automated melting point apparatus EZ-MELT and were given without correction.

1. **Synthetic procedures**

Compounds **1p** and **1m** were synthesized according to reported procedures.^1,2^

**Supplementary Scheme 1.** The preparation for compounds **1o** and **2p**,**m**,**o**.

**2,5-*bis*(2-nitrophenyl)-1,4-bis(4-octylphenyl)-1,4-dihydropyrrolo[3,2-*b*]pyrrole (1o).** 2-Nitrobenzal-dehyde (0.76 g, 5 mmol), 4-octylaniline (1.03 g, 5 mmol), and *p*-toluenesulfonic acid (0.085 g, 0.5 mmol) in 5 mL of glacial acetic acid were heated at 90°C for 30 min with stirring. Butane-2,3-dione (0.22 g, 0.22 mL, 2.5 mmol) was then slowly added and the resulting mixture was stirred at 90 °C for additional 3 h. On cooling the precipitated product was filtered and washed with cooled glacial acetic acid. Recrystallization from AcOH gave 0.73 g (40%) of pure entitled product. M.p. 176^o^C. ^1^H NMR (500 MHz, CDCl_3_) *δ* 7.72 (dd, *J* = 8.2, 1.3 Hz, 2H), 7.48 (td, *J* = 7.5, 1.3 Hz, 2H), 7.42 (dd, *J* = 7.8, 1.5 Hz, 2H), 7.35 (td, *J* = 7.3, 1.4 Hz, 2H), 7.09 (s, 8H), 6.35 (s, 2H), 2.58 (t, *J* = 7.8 Hz, 4H), 1.61 (quint, *J* = 7.5 Hz, 4H), 1.35 – 1.23 (m, 20H), 0.88 (t, *J* = 7.0 Hz, 6H); ^13^C NMR (126 MHz, CDCl_3_) *δ* 149.0, 140.7, 136.3, 133.0, 132.0, 131.5, 130.4, 129.1, 128.3, 127.7, 124.5, 124.1, 95.3, 35.4, 31.9, 31.3, 29.4, 29.3, 29.2, 22.7, 14.1 ppm; HRMS (EI) calcd for C_46_H_52_N_4_O_4_: 724.3989 [M^+∙^], found: 724.3998.

**General procedure for the preparation of compounds 2p, 2m, and 2o.** Aromatic aldehyde (3 mmol) and 4-octylaniline (0.62 g, 3 mmol) were heated in neat at 120-130^o^C for 2 hours. Butan-2,3-dione (0.13 g, 0.13 mL, 1.5 mmol), dry *p*-toluenesulfonic acid (0.05 g, 0.3 mmol) and 5 mL of glacial acetic acid were added to cooled to 90^o^C mixture. The reaction was heated at 90^o^C for additional 4 h. On cooling the precipitated product was filtered and washed with acetic acid.

**2,5-bis(4'-nitro-[1,1'-biphenyl]-4-yl)-1,4-bis(4-octylphenyl)-1,4-dihydropyrrolo[3,2-*b*]pyrrole (2p).** The crude product was subsequently washed with diethyl ether and acetonitrile and then recrystallized from ethyl acetate. Yield 31%. M.p. 237-238^o^C. ^1^H NMR (500 MHz, CDCl_3_) *δ* 8.26 (d, *J* = 8.4 Hz, 4H), 7.71 (d, *J* = 8.5 Hz, 4H), 7.49 (d, *J* = 8.0 Hz, 4H), 7.34 (d, *J* = 8.0 Hz, 4H), 7.27 – 7.23 (m, 4H), 7.20 (d, *J* = 8.0 Hz, 4H), 6.48 (s, 2H), 2.65 (t, *J* = 7.8 Hz, 4H), 1.66 (br. s, 4H), 1.40 – 1.19 (m, 20H), 0.88 ppm (m, 6H); ^13^C NMR (126 MHz, CDCl_3_) *δ* 147.1, 146.9, 141.0, 137.5, 135.8, 135.4, 134.4, 132.6, 129.2, 128.4, 127.3, 127.0, 125.2, 124.1, 95.0, 35.5, 31.9, 31.3, 29.5, 29.3 (2 signals), 22.7, 14.1 ppm; HRMS (EI) calcd for C_58_H_60_N_4_O_4_: 876.4615 [M^+·^], found: 876.4636.

**2,5-bis(3'-nitro-[1,1'-biphenyl]-4-yl)-1,4-bis(4-octylphenyl)-1,4-dihydropyrrolo[3,2-*b*]pyrrole (2m).** The product was subsequently washed with diethyl ether and acetonitrile. Yield 17%. M.p. 181-182^o^C. ^1^H NMR (600 MHz, CDCl_3_) *δ* 8.43 (t, *J* = 2.0 Hz, 2H), 8.15 (ddd, *J* = 8.2, 2.3, 1.0 Hz, 2H), 7.89 (ddd, *J* = 7.8, 1.8, 1.0 Hz, 2H), 7.57 (t, *J* = 8.0 Hz, 2H), 7.49 (d, *J* = 8.5 Hz, 4H), 7.34 (d, *J* = 8.5 Hz, 4H), 7.26 – 7.23 (m, 4H), 7.20 (d, *J* = 8.4 Hz, 4H), 6.47 (s, 2H), 2.64 (t, *J* = 7.7 Hz, 4H), 1.65 (quint, *J* = 7.7 Hz, 4H), 1.39 – 1.23 (m, 20H), 0.87 (t, *J* = 7.1 Hz, 6H); ^13^C NMR (151 MHz, CDCl_3_) *δ* 148.8, 142.4, 140.9, 137.5, 135.8, 135.3, 134.0, 132.6, 132.4, 129.6, 129.2, 128.5, 126.8, 125.2, 121.8, 121.6, 94.9, 35.5, 31.9, 31.3, 29.5, 29.4, 29.3, 22.7, 14.1 ppm; HRMS (EI) calcd for C_58_H_60_N_4_O_4_: 876.4615 [M^+·^], found: 876.4634.

**2,5-bis(2'-nitro-[1,1'-biphenyl]-4-yl)-1,4-bis(4-octylphenyl)-1,4-dihydropyrrolo[3,2-*b*]pyrrole (2o).** The product was washed with acetonitrile. Yield 5.2%. M.p. 153^o^C. ^1^H NMR (600 MHz, CDCl_3_) *δ* 7.81 (dd, *J* = 8.4, 1.3 Hz, 2H), 7.58 (td, *J* = 7.6, 1.3 Hz, 2H), 7.46 – 7.42 (m, 4H), 7.27 (d, *J* = 8.5 Hz, 4H), 7.22 (d, *J* = 8.5 Hz, 4H), 7.19 (d, *J* = 8.6 Hz, 4H), 7.17 (d, *J* = 8.5 Hz, 4H), 6.45 (s, 2H), 2.63 (t, *J* = 7.7 Hz, 4H), 1.64 (quint, *J* = 7.7 Hz, 4H), 1.38 – 1.22 (m, 20H), 0.87 (t, *J* = 7.0 Hz, 6H); ^13^C NMR (151 MHz, CDCl_3_) *δ* 149.3, 140.6, 137.5, 136.0, 135.2, 134.6, 133.7, 132.2, 132.1, 131.8, 129.1, 128.1, 127.9, 127.7, 125.1, 124.1, 94.9, 35.5, 31.9, 31.3, 29.5, 29.3 (2 signals), 22.7, 14.1 ppm; HRMS (EI) calcd for C_58_H_60_N_4_O_4_: 876.4615 [M^+·^], found: 876.4592.

1. **^1^H and ^13^C NMR spectra for synthesized compounds**


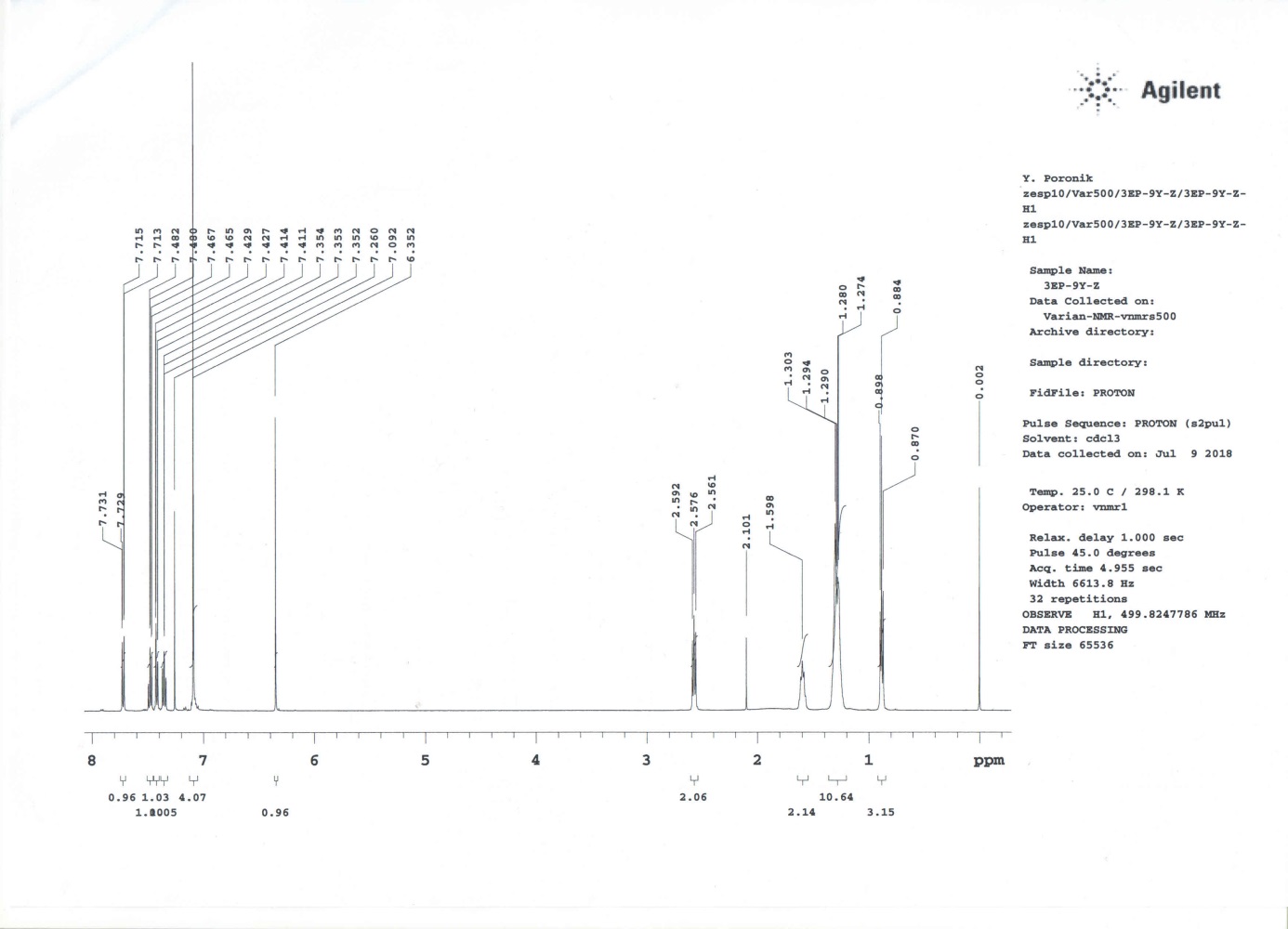


**Supplementary Figure 1.** ^1^H NMR spectrum of **1o**.


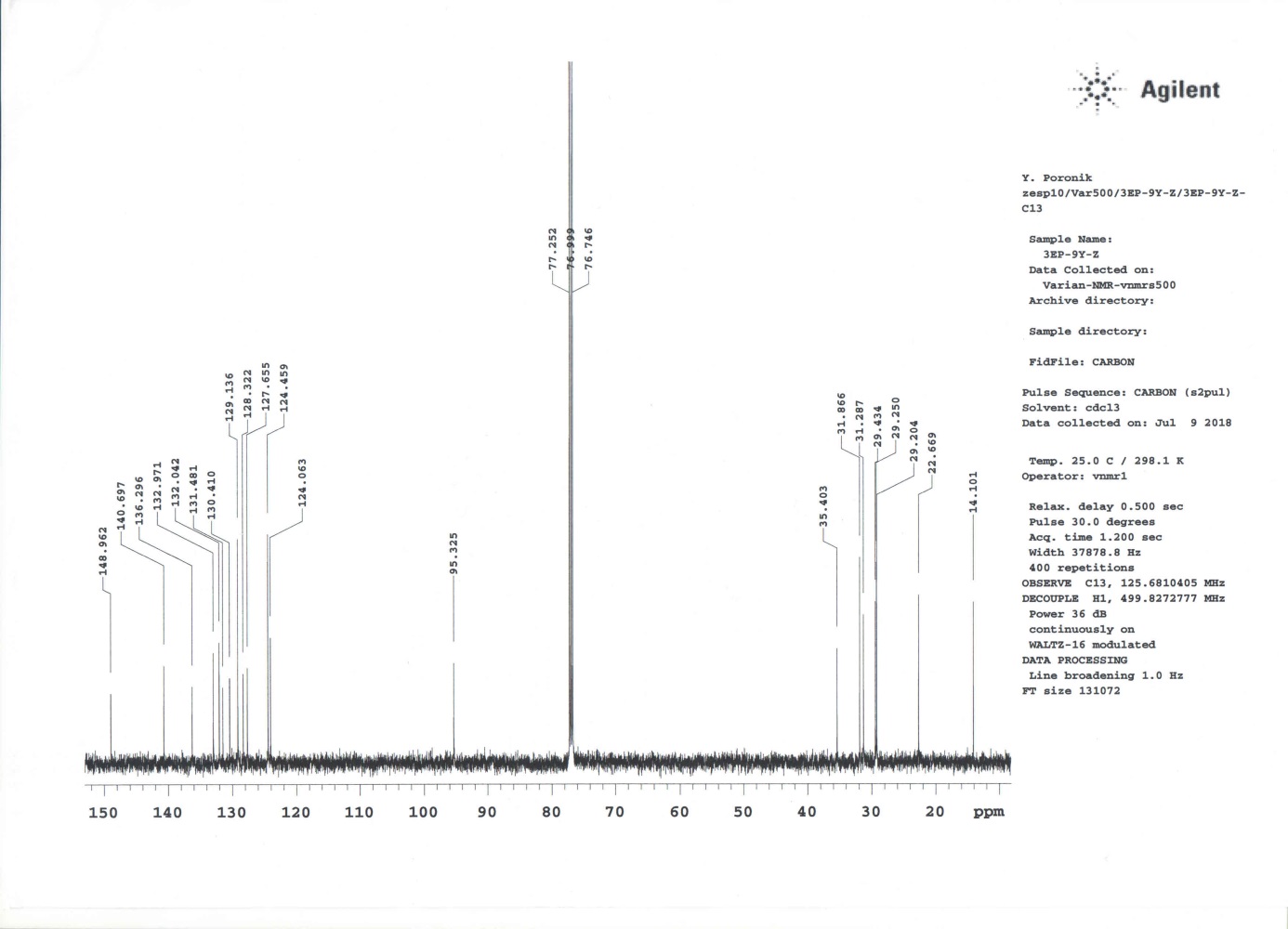


**Supplementary Figure 2.** ^13^C NMR spectrum of **1o**.


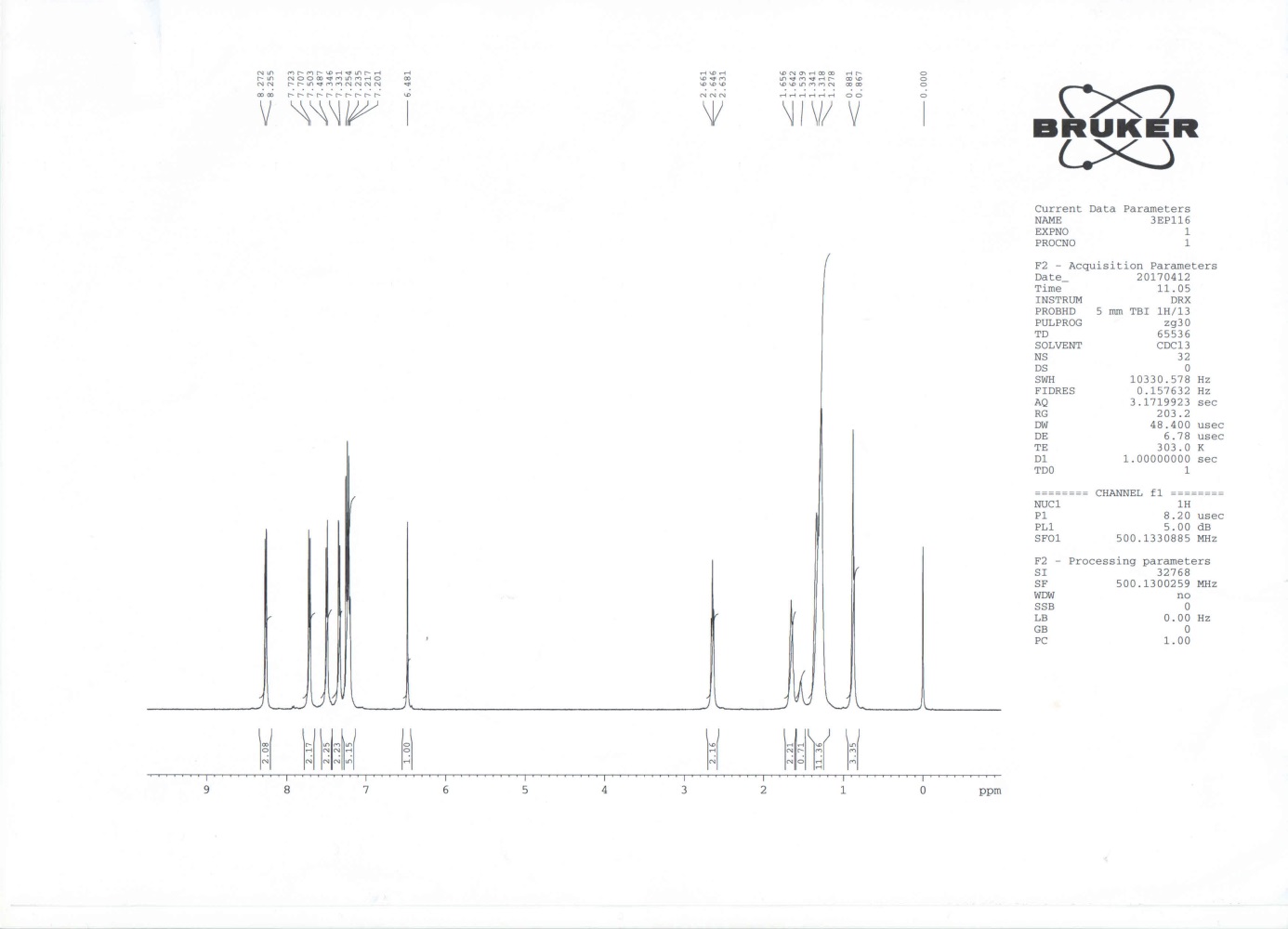


**Supplementary Figure 3.** ^1^H NMR spectrum of **2p**.


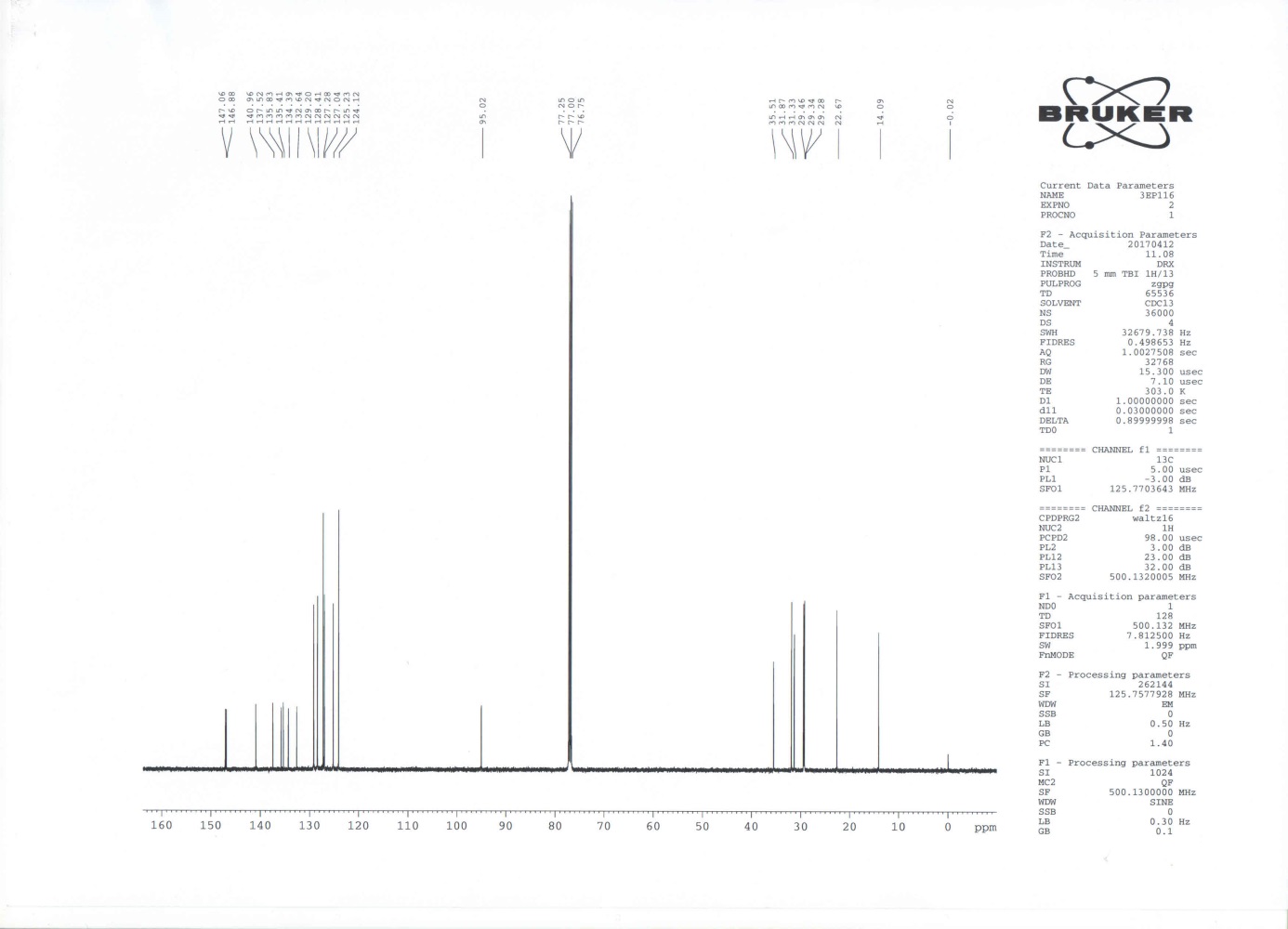


**Supplementary Figure 4.** ^13^C NMR spectrum of **2p**.


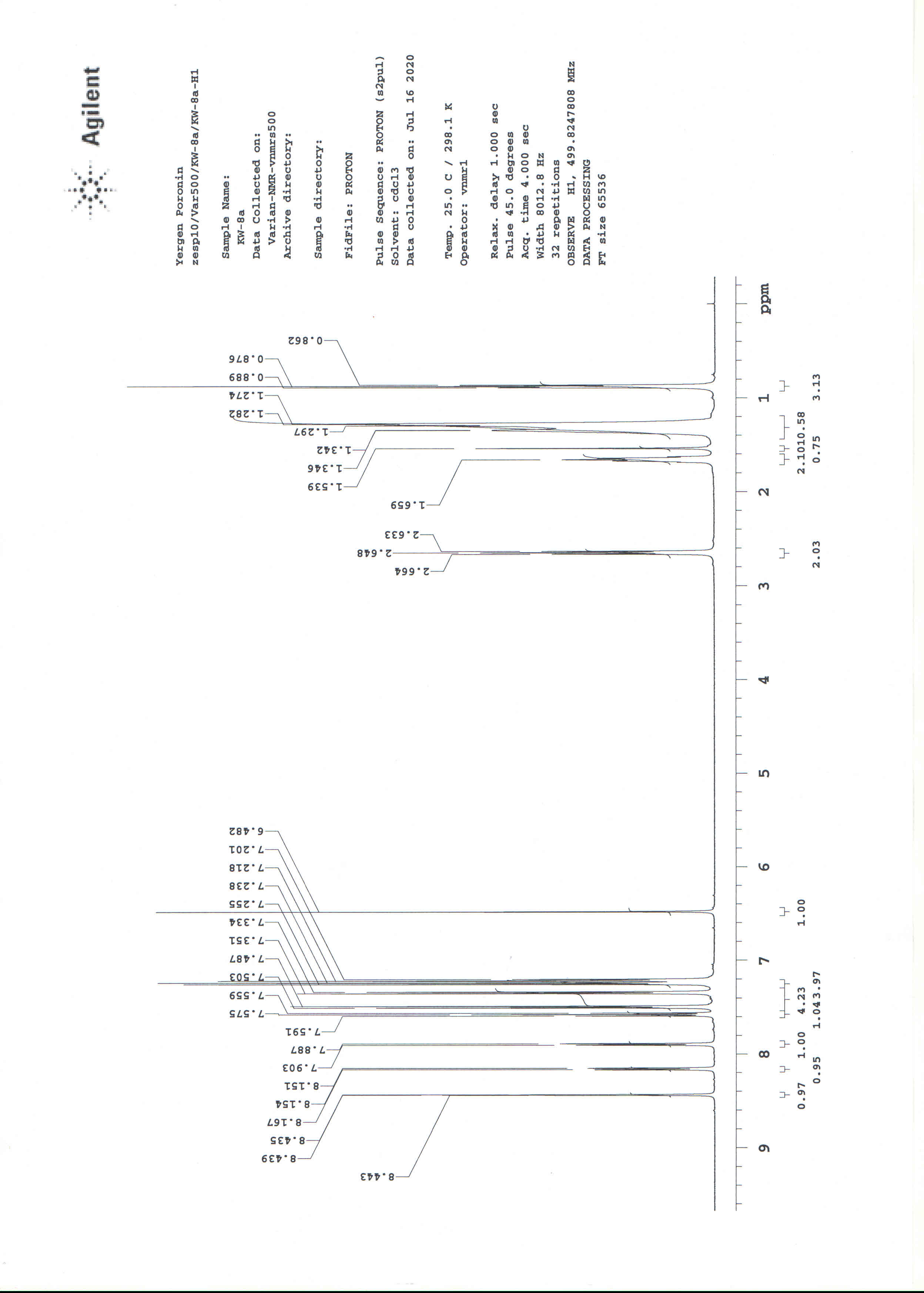


**Supplementary Figure 5.** ^1^H NMR spectrum of **2m**.


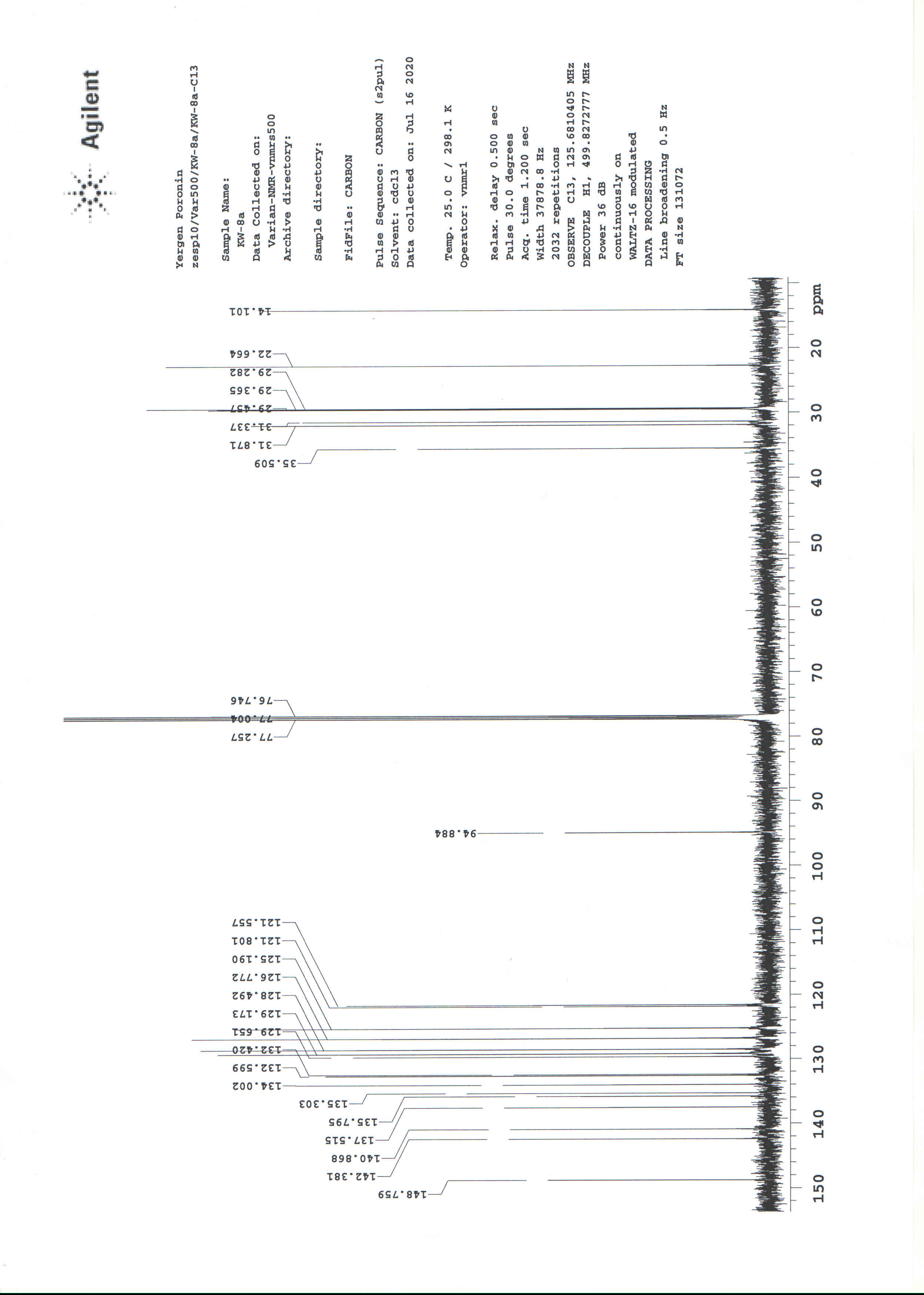


**Supplementary Figure 6.** ^13^C NMR spectrum of **2m**.


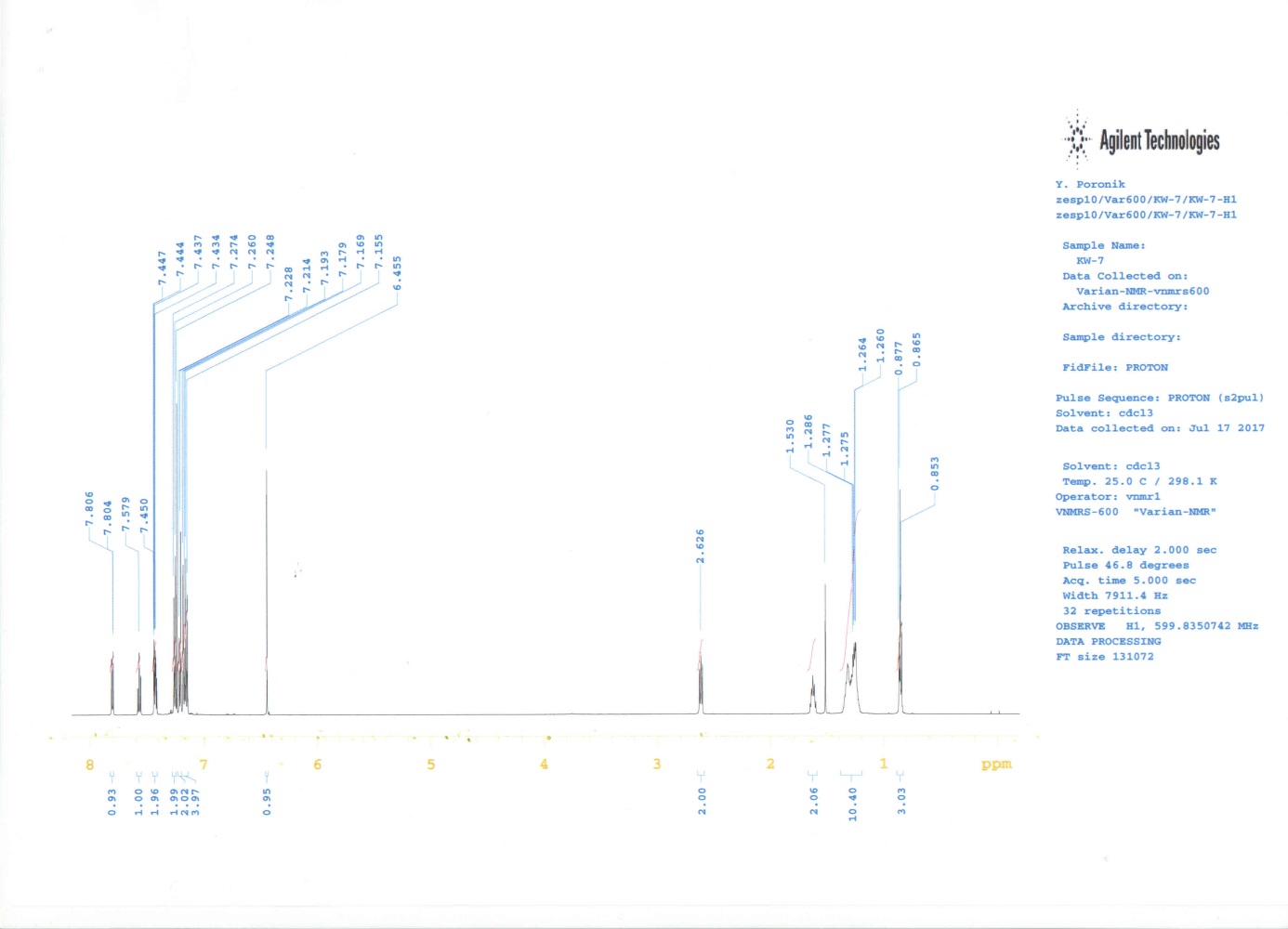


**Supplementary Figure 7.** ^1^H NMR spectrum of **2o**.


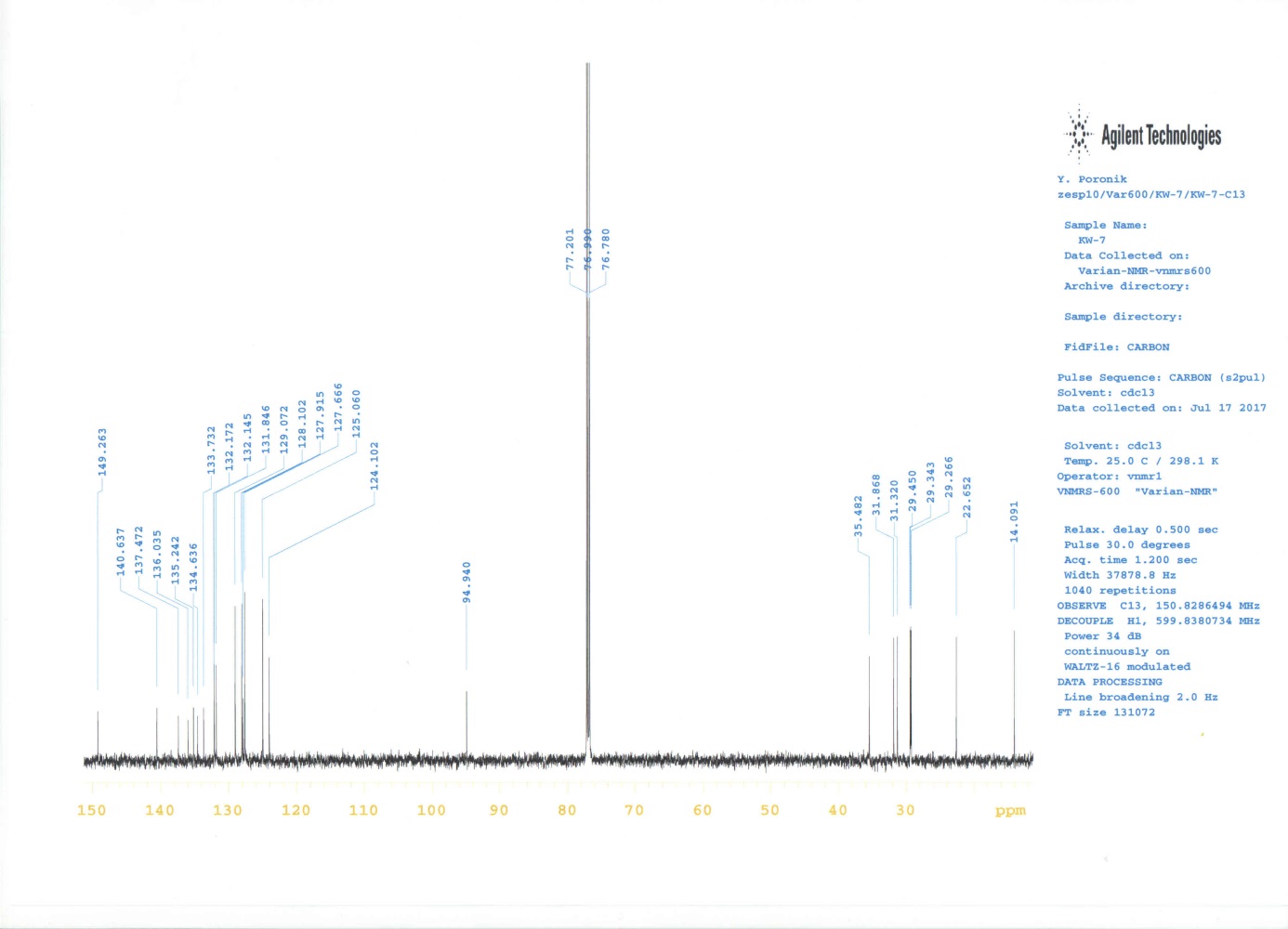


**Supplementary Figure 8.** ^13^C NMR spectrum of **2o**.

1. **Steady-state absorption and emission.**

A Perkin-Elmer Lambda 25 UV/Vis spectrophotometer and a Hitachi F7000 fluorescence spectrometer were used to acquire steady-state absorption and emission spectra respectively. Spectroscopic grade solvents were used without further purification. All photophysical studies have been performed with freshly-prepared air-equilibrated solutions at room temperature (298 K).

The concentration dependence of the absorbance allows for estimating the molar extinction coefficients, *ε*, and the difference between absorption and emission maxima yields the Stokes’ shifts, Δ*S* (Supplementary Table 1)

Relative fluorescence quantum yields are calculated using the steady-state data (Supplementary Table 1) on the basis of the reported protocol.^3,4^ Fluorescein in 0.1N solution of NaOH in water and 9,10-diphenylanthracene in cyclohexane are used as the standards.

*Preparation of SOA solutions for measurements:* Concentrated dye solutions in ethyl acetate is added to a homogeneous mixture of SOA and ethyl acetate 9:1 (small amount of ethyl acetate makes the mixture less viscous). A homogenous dye solution is concentrated at reduced pressure and viscous foam-like dye solution in SOA was additionally dried in high-vacuum for several hours. The flask with the SOA solution was immersed into an oil bath (100-110^o^C) and as soon as it became liquid it is rapidly transferred into the cuvette. To get rid of air bubbles, the cuvette was put to a round-bottom flask and the flask was immersed to the oil bath for several minutes. After cooling, the cuvette with the sample is ready for measurements.

**Supplementary Table 1.** Absorption and fluorescence properties for compounds **1p,m,o** and **2p,m,o**.

|  | solvent | *λ*_abs_ [nm] | *ε*×10^3^ [M^-1^cm^-1^] | *λ*_fl_ [nm] | Δ$\tilde{\nu}$ [cm^-1^] | *ϕ_f_* |
| --- | --- | --- | --- | --- | --- | --- |
| **1p**^a^ | C_6_H_12_ | 447; 469 | 48; 44 | 496, 525 | 1200 | 0.96 |
|  | CCl_4_ | 458 | 45 | 540 | 4100 | 0.93 |
|  | toluene | 465 | 42 | 552; 569 | 4200 | 0.70 |
|  | dioxane | 464 | 44 | 590 | 5200 | 0.25 |
|  | MTBE | 458 | 41.5 | 565 | 5000 | 0.42 |
|  | THF | 471 | 44 | 610 | 5800 | 0.03 |
|  | anisole | 476 | 36 | 627 | 5700 | 0.05 |
|  | CHCl_3_ | 477 | 41.5 | 660 | 6900 | 0.0027 |
|  | CH_2_Cl_2_ | 477 | 41 | nd |  |  |
|  | CH_3_CN | 467 | 40 | nd |  |  |
|  | DMSO | 483 | 35 | nd |  |  |
|  | EtOAc | 464 |  | 614 | 6000 | 0.02 |
|  | SOA^d^ | 472 |  | 580 | 4800 | 0.59 |
| **1m** ^b^ | C_6_H_12_ | 359 | 39 | 533 | 10100 | 0.0383 |
|  | CCl_4_ | 363 | 35 | 626 | 12500 | 0.0045 |
|  | toluene | 362 | 39 | 650 | 12600 | 0.0014 |
|  | MTBE | 358 | 40 | 661 | 14500 | 0.0015 |
|  | CH_2_Cl_2_ | 360 | 40 | nd |  |  |
|  | DMSO | 363 | 40 | nd |  |  |
| **1o** | C_6_H_12_ | 419; 335; 285 | 6; 21; 21 | nd |  |  |
|  | CCl_4_ | 424; 338; 298 | 6; 20; 21 | nd |  |  |
|  | toluene | 428; 335; 298 | 6; 22; 21 | nd |  |  |
|  | CH_2_Cl_2_ | 436; 331; 299 | 5; 22; 23 | nd |  |  |
|  | DMSO | 435; 330; 300 | 5; 23; 24 | nd |  |  |
| **2p**^c^ | C_6_H_12_ | 439 | 50 | 513 | 4300 | 1.04 |
|  | CCl_4_ | 446 | 42 | 577 | 6100 | 0.14 |
|  | toluene | 444 | 41 | 610 | 6700 | 0.33 |
|  | dioxane | 442 | 45 | 653 | 8100 | 0.082 |
|  | MTBE | 435 | 39 | 655 | 8400 | 0.063^b^ |
|  | THF | 446 | 43 | 708 | 8300 | 0.0023 |
|  | anisole | 448 | 39 | 706 | 8800 | 0.006^b^ |
|  | CH_2_Cl_2_ | 444 | 38 | nd |  |  |
|  | CH_3_CN | 439 | 26 | nd |  |  |
|  | DMSO | 451 | 31 | nd |  |  |
|  | EtOAc | 437 |  | 717 | 9300 | 0.02 |
|  | SOA^d^ | 442 |  | 628 | 7200 | 0.15 |
| **2m**^b^ | C_6_H_12_ | 395 | 48 | 507 | 5900 | 0.12 |
|  | CCl_4_ | 399 | 46 | 620 | 9900 | 0.01 |
|  | toluene | 395 | 48 | 648 | 10500 | 0.005 |
|  | dioxane | 394 | 51.5 | nd |  |  |
|  | MTBE | 392 | 48 | 717 | 12200 | 0.0009 |
|  | THF | 396 | 51 | nd |  |  |
|  | CH_2_Cl_2_ | 392 | 46 | nd |  |  |
|  | CH_3_CN | 387 | 47 | nd |  |  |
|  | DMSO | 396 | 47 | nd |  |  |
| **2o**^b^ | C_6_H_12_ | 369 | 36 | 438 | 4300 | 0.0006 |
|  | toluene | 372 | 36 | 438 | 4100 | 0.0007 |
|  | dioxane | 371 | 39 | 440 | 4200 | 0.0007 |
|  | THF | 372 | 38.5 | 440 | 4200 | 0.0005 |
|  | CH_2_Cl_2_ | 369 | 39 | 444 | 4600 | 0.0004 |
|  | CH_3_CN | 365 | 39 | 440 | 4700 | 0.0003 |
|  | DMSO | 373 | 38 | 457 | 4900 | 0.0003 |

^a^ – Fluorescein in 0.1N NaOH solution in water was used as the fluorescence standard, *λ*_ex_ = 464 nm. In C_6_H_12_ and CCl_4_ for **1p**, *λ*_ex_ = 460 nm;

^b^ – 9,10-Diphenylanthracene in cyclohexane was used as the fluorescence standard, *λ*_ex_ = 373 nm. For **1m**, *λ*_ex_ = 347 nm;

^c^ – Coumarin 153 in ethanol was used as the fluorescence standard, *λ*_ex_ = 440 nm;

^d^ – Measured refraction index for SOA: *n_D_* = 1.466.

1. **Computational details**

At the first stage the ground singlet state (S0) molecular geometries of the compounds **1p**, **1m** and **1o** have been optimized by the B3LYP/6-31+G(d) method in a gas phase approximation. At the next stage, based on the equilibrium S_0_ geometries the S_1_ and T_1_ excited states have been optimized at the TD-DFT and unrestricted DFT levels of theory using the same B3LYP/6-31+G(d) gas phase approach.^5-9^ We have found, that B3LYP functional significantly underestimates the energy of the S_1_ state for all the TAPPs **1p**,**m,o** and **2p,m,o** (here we mean the energy of relaxed S_1_ state responsible for the fluorescence). Applying the PCM solvation model^10^ does not improve the S_1_ state energies considerably, thus we can conclude that underestimation effect relates mainly to the inner limitation of B3LYP functional in respect to the space-separated charge transfer states. That is why, the range separated CAM-B3LYP functional^11^ was future used for analysis of photophysical constants of **1p**,**m,o** and **2p,m,o** molecules.

At initial stage we optimized the S_1_ and T_1_ states of all studied dyes by using TDDFT/B3LYP and UB3LYP methods. Usually, S_1_ and T_1_ are characterized by a similar geometry because of they are usually of the same electronic configuration (HOMO-LUMO type in most cases). In vertical absorption spectra (starting from S_0_ ground state geometry) we really see that S_1_ and T_1_ are of the same configuration, but optimized S_1_ and T_1_ states are very different in structure. T_1_ state geometry is closer to the ground state conformation, while S_1_ global minimum (TICT) demonstrate a strong rotation along D-A pairs and not demonstrate a fluorescence. Thus, we decided that it should be additional “bright” local minimum on S_1_ PES. Starting from T_1_ state geometry and optimizing it by TDDFT/B3LYP we obtained S_1_(CT) bright state which is very close to T_1_ state geometry and demonstrate a strong fluorescence. In all cases (T_1_, S_1_^(CT)^, S_1_^(TICT)^) we checked the vibrational spectra of the optimized structures. No imaginary frequencies were found meaning that found geometries correspond to the genuine PES minima.

Starting from the S_1_^(CT)^ and S_1_^(TICT)^ optimized excited state geometries the spin-orbit coupling (SOC) effects were treated as a perturbation based on the scalar relativistic (SR) orbitals after SCF and TDDFT calculations (pSOC-TDDFT);^12^ CAM-B3LYP functional, Slater-type DZP all-electron basis set^13^ and COSMO continuum solvation model (hexane as a solvent)^14^ were used for these calculations. The SOC matrix elements, $\left\langle S_{1} | \hat{H}_{\mathrm{SO}} | T_{j} \right\rangle$ (*j* = 1,2,3…; *E*(S_1_) > *E*(T*_j_*)) were calculated as root mean squares, i.e. as square root of the sum of squares of spin-orbit coupling matrix elements of all triplet state sublevels (*m*=0,±1) of the uncoupled states:^15^

|  | $\left\langle S_{i} \vert\hat{H}_{\mathrm{SO}} \vert T_{j} \right\rangle=\sqrt{\sum_{m=0, \pm1} \left\langle S_{i} \vert\hat{H}_{\mathrm{SO}} \vert T_{j}^{m} \right\rangle^{2}}$ | (S1) |
| --- | --- | --- |

The spin-orbit coupling operator $\hat{H}_{\mathrm{SO}}$ was considered in our calculations within the zeroth-order regular approximation (ZORA)^16,17^ in accordance with the following expression:

|  | $\hat{H}_{\mathrm{SO}}=\frac{c^{2}}{{\left( {2c}^{2} \right.-\left. V \right)}^{2}}\sigma\left( \nabla V \right.\cdot\left. p \right),$ | (S2) |
| --- | --- | --- |

where **σ** – Pauli spin matrix vector, **p** – the linear momentum operator; *c* – speed of light, *V*– Kohn−Sham potential. The fluorescence rate constants (*k_r_*) were estimated according to the following relationship (expressed in atomic units):^18,19^

|  | $k_{r}=\frac{1}{\tau}=\frac{2\left( {\Delta E}^{2} \right)f}{c^{3}},$ | (S3) |
| --- | --- | --- |

where *τ* is radiative life of S_1_ state,
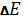
 and *f –* the energy and intensity of the corresponding singlet-singlet or singlet-triplet transitions with accounting of SOC perturbations.

The rate constants of intersystem crossing (ISC) between the S_1_ and T*_j_* states E(S_1_) > E(T*_j_*) were estimated using the Plotnikov’s simple empirical approximation:^20^

|  | $k_{S_{1}\to T_{j}}={10}^{10}\left\langle S_{1} \vert\hat{H}_{\mathrm{so}} \vert T_{j} \right\rangle^{2}F_{0m},$ | (S4) |
| --- | --- | --- |

where Franck–Condon factors (*F*_0m_) were approximated using the formula:

|  | $F_{0m}=\sum_{n} \prod_{\nu} \frac{e^{-y}y^{n_{\nu}}}{n_{\nu}!}$ | (S5) |
| --- | --- | --- |

In Supplementary Equation (S5) Huang–Rhys factor *y* was assumed to be equal to 0.3 and only one average promotive mode *ω_ν_* = 1400 cm^-1^ was used when considering $n_{\nu}=\Delta E\left( S_{1} \right.-\left. T_{j} \right){/\omega}_{\nu}$. Such a single-mode approximation was considered efficient and accurate enough for the organic dyes and fluorophores.^21-24^

The internal conversion (IC) rate calculations were performed within the both Franck–Condon and Herzberg–Teller approaches^25-27^ using the non-adiabatic coupling matrix element between the S_1_ and S_0_ states calculated at the TDDFT/B3LYP/6-31+G(d) level of theory.

The pSOC-TDDFT calculations were carried out using the ADF2018 package^28^ while the rest of calculations were performed using Gaussian16 software.^29^


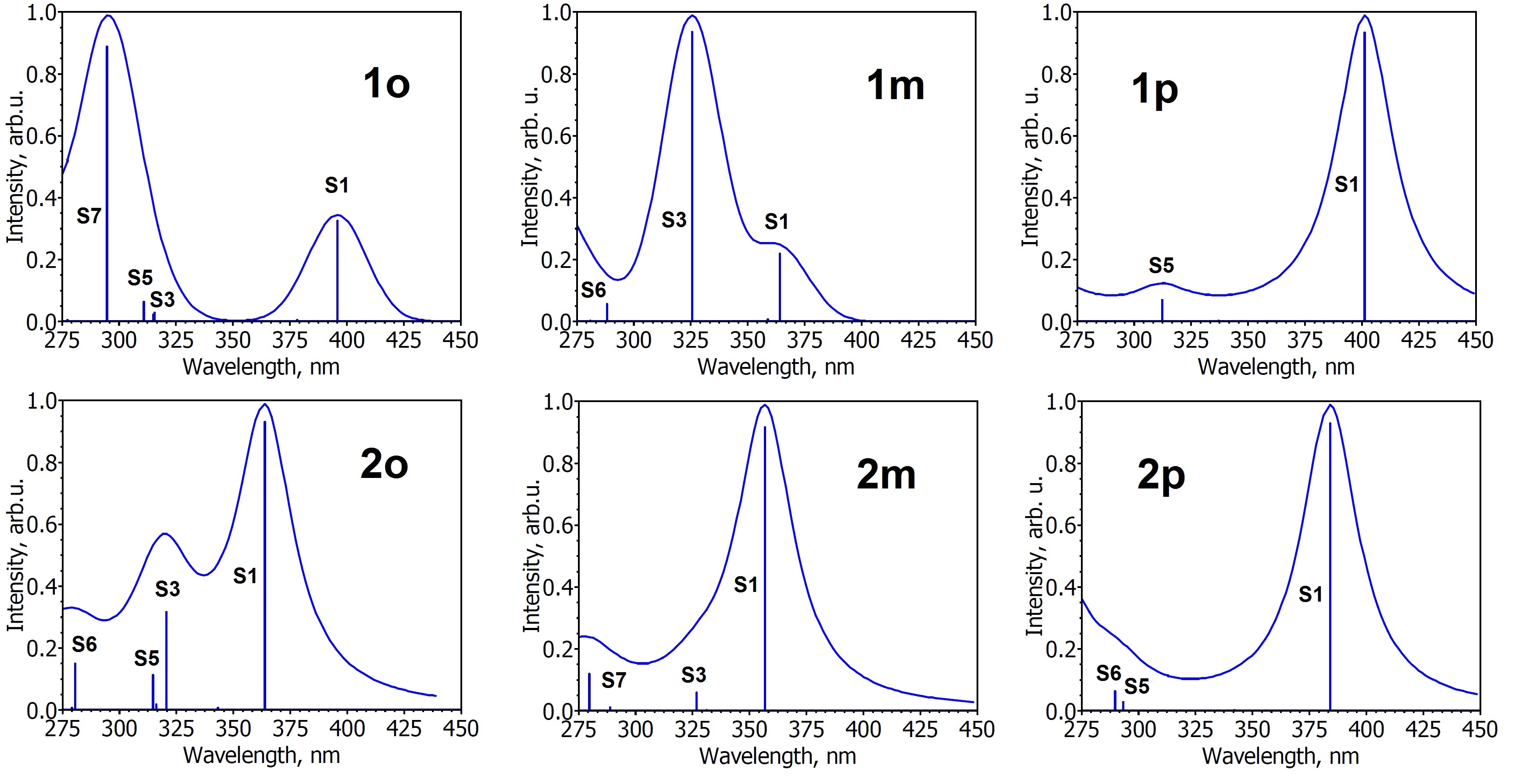


**Supplementary Figure 9.** Absorption spectra of TAPPs **1o**, **1m**, **1p, 2o**, **2m** and **2p** calculated by TDDFT/B3LYP/6-31+G(d) method with accounting of solvent effect (hexane). Excited state assignments are presented for most intense transitions.

|  | **1p** | | | **2p** | | | | | | |
| --- | --- | --- | --- | --- | --- | --- | --- | --- | --- | --- |
|  | **Steady state**  **geometry** | **CT** | **TICT** | **Steady state**  **geometry** | | **CT** | **TICT** | | | |
| **LUMO** | 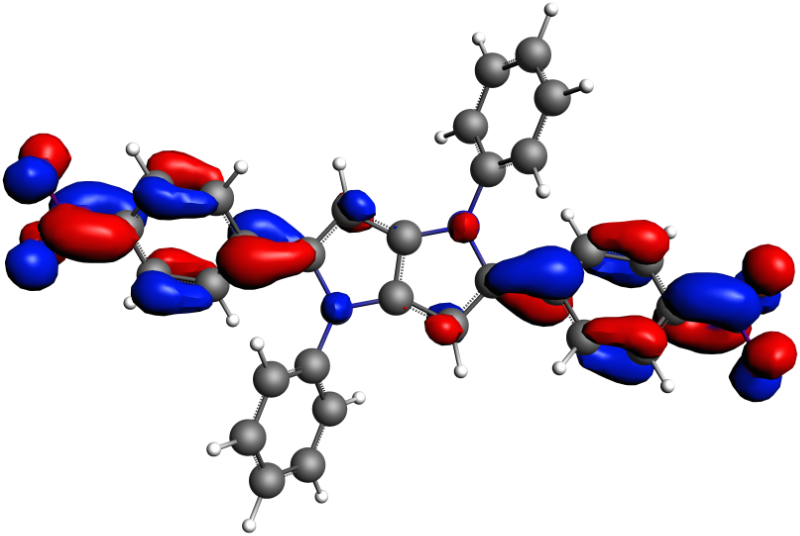 | 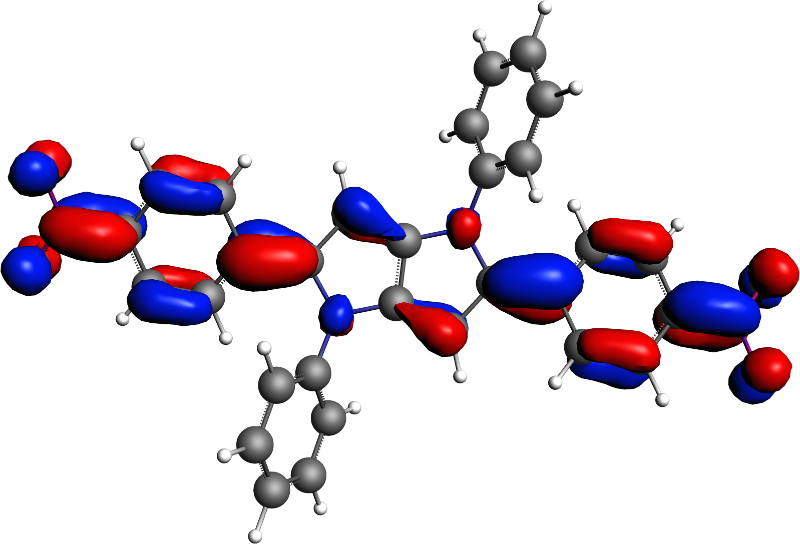 | 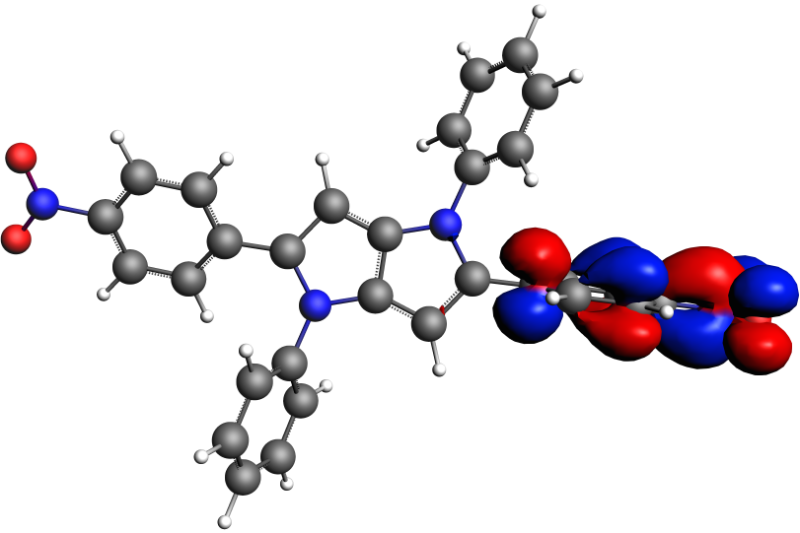 | 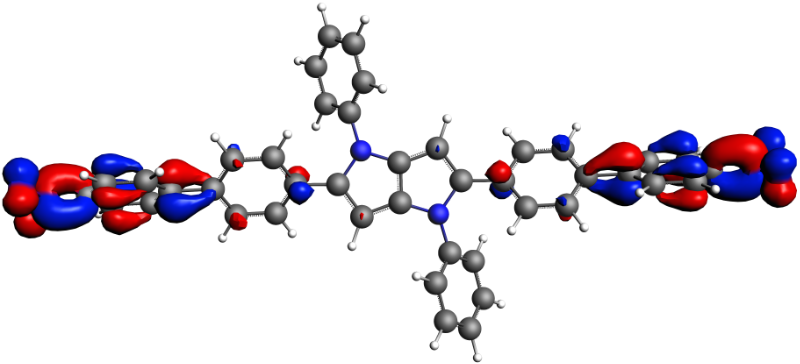 | | 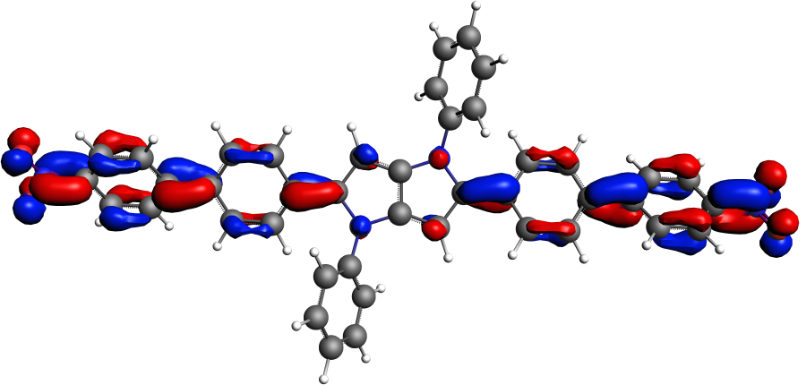 | | 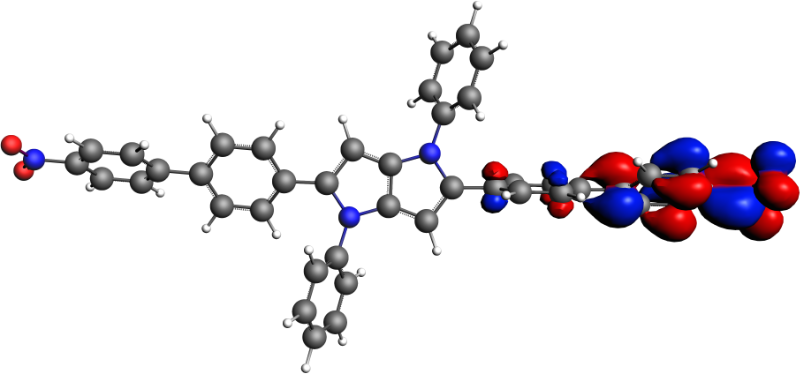 | | | |
| **HOMO** | 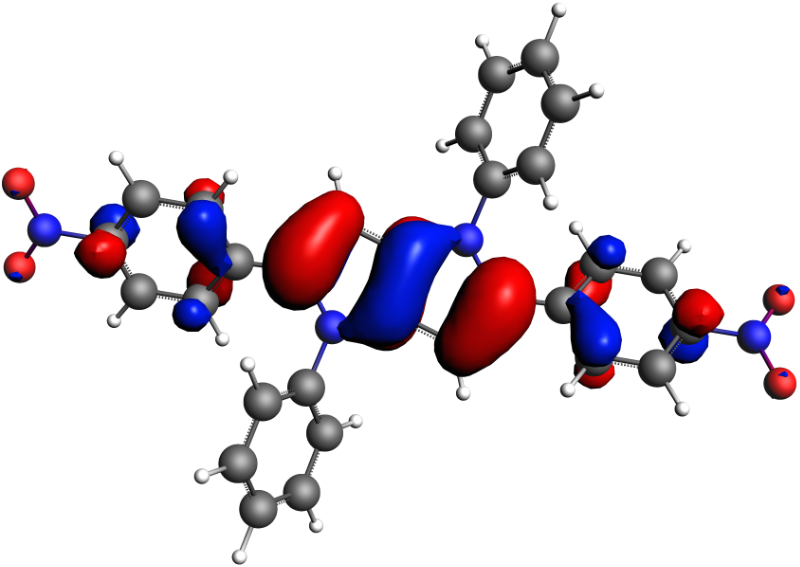 | 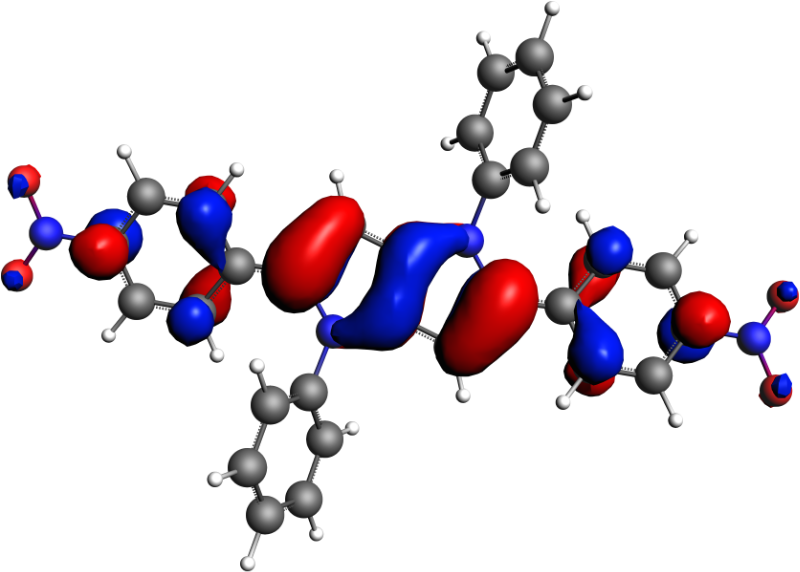 | 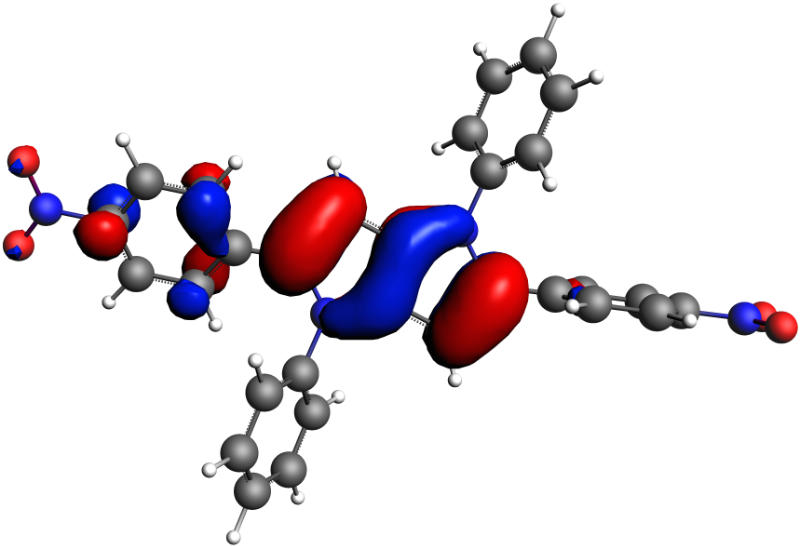 | 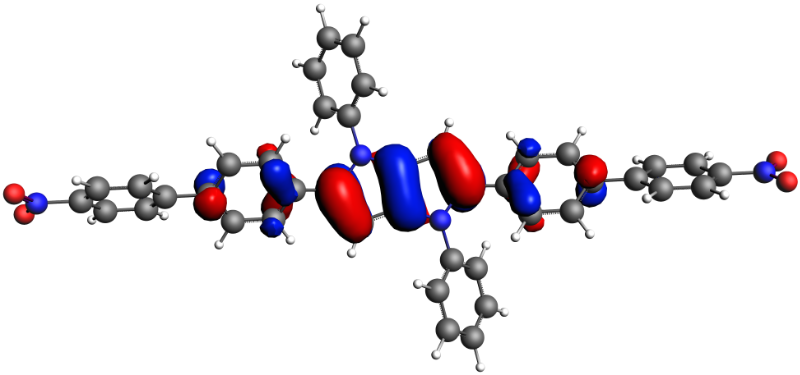 | | 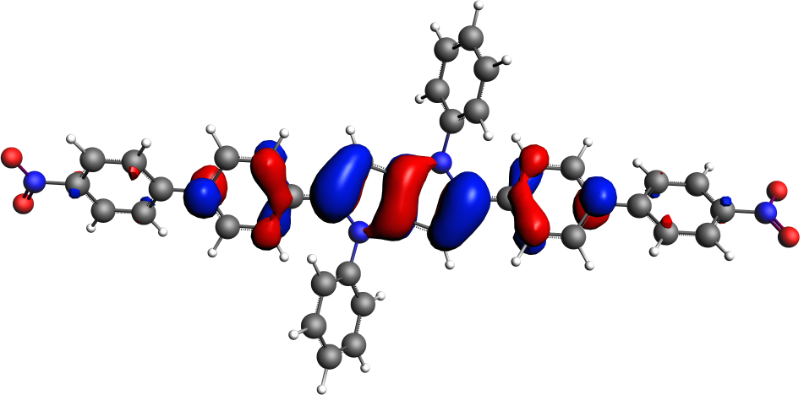 | | 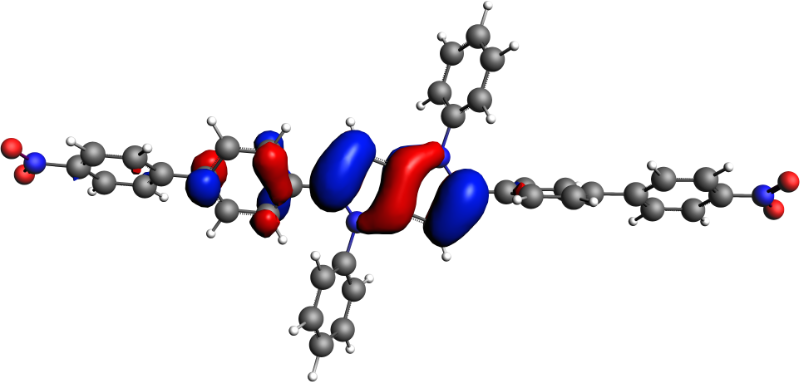 | | | |
|  | **1m** | | | **2m** | | | | | | |
| **LUMO** | 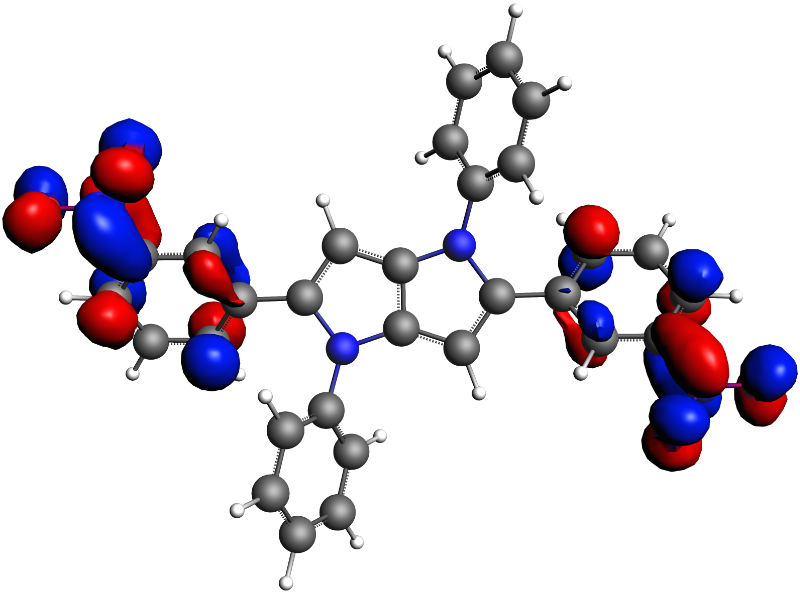 | 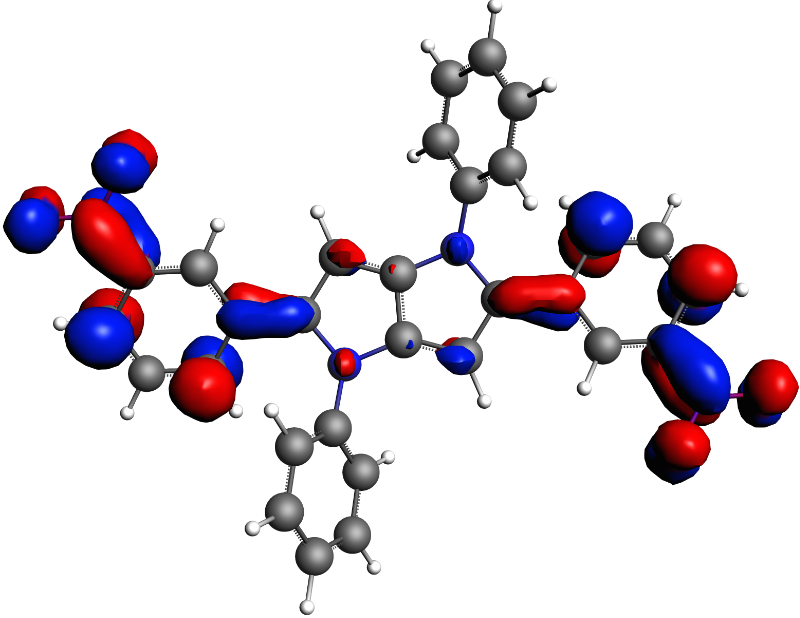 | 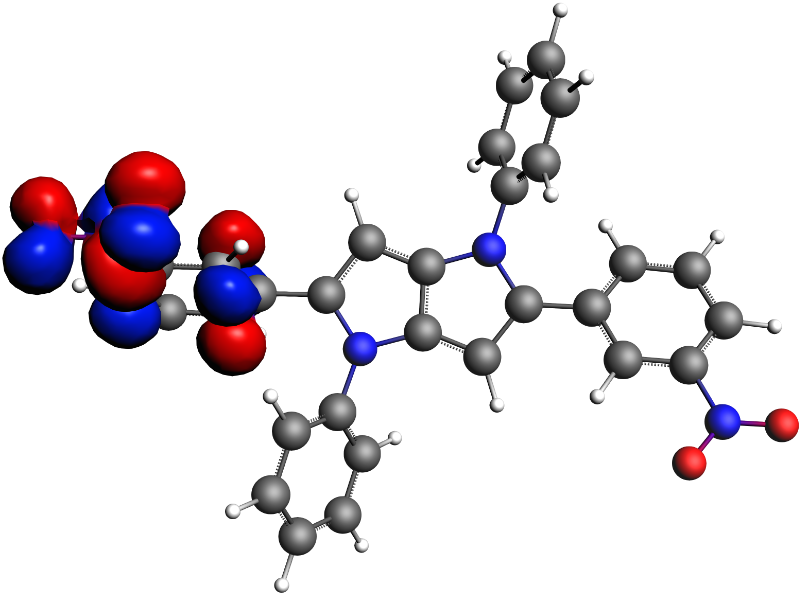 | 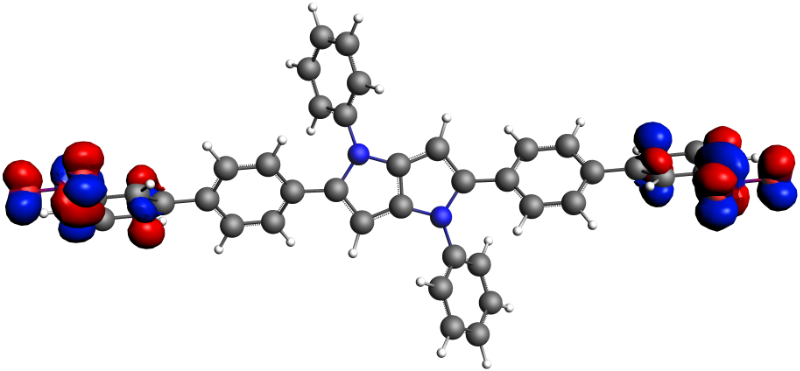 | 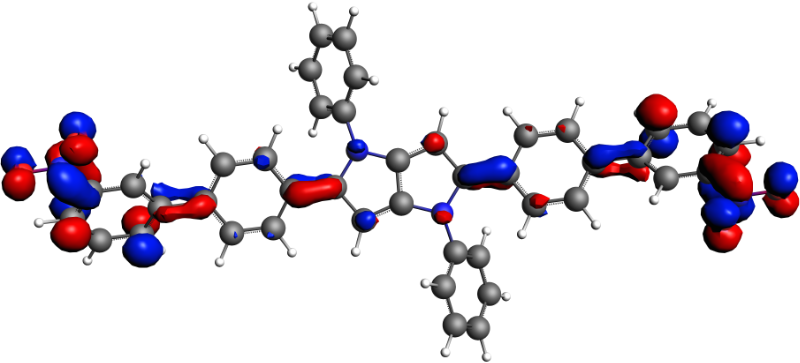 | | | | 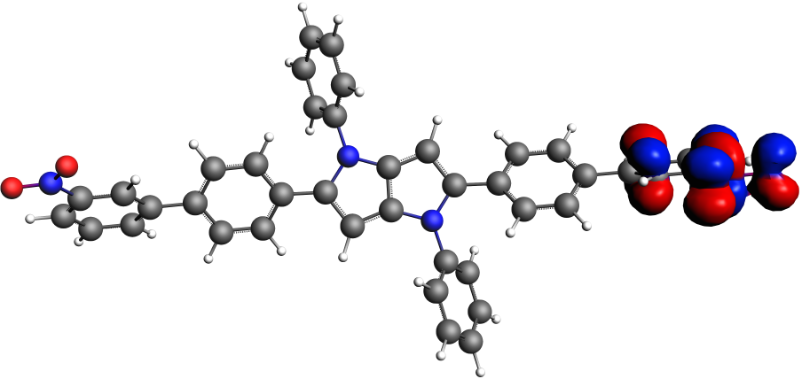 | | |
| **HOMO** | 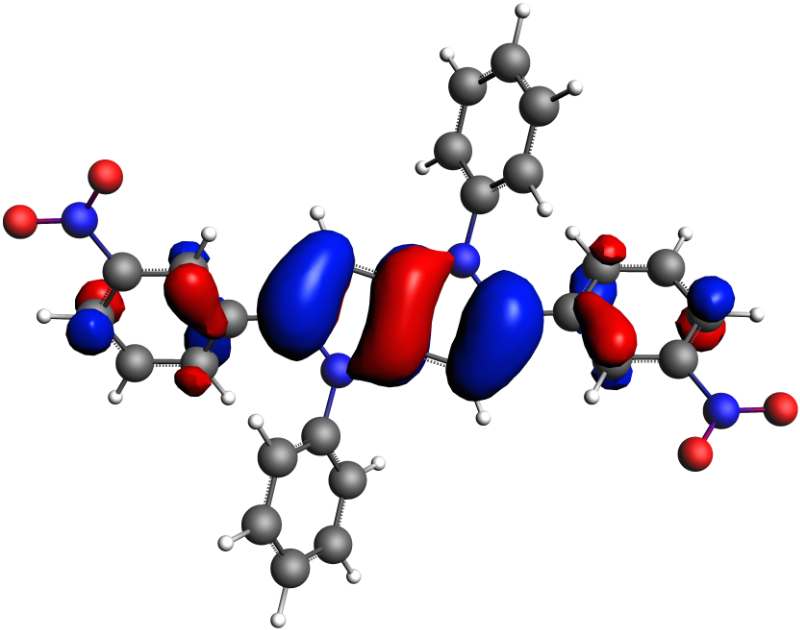 | 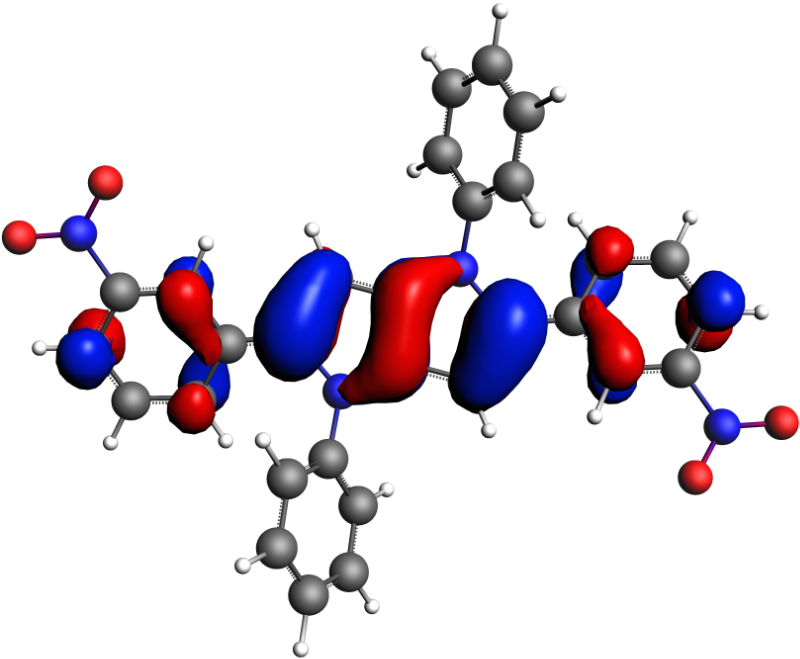 | 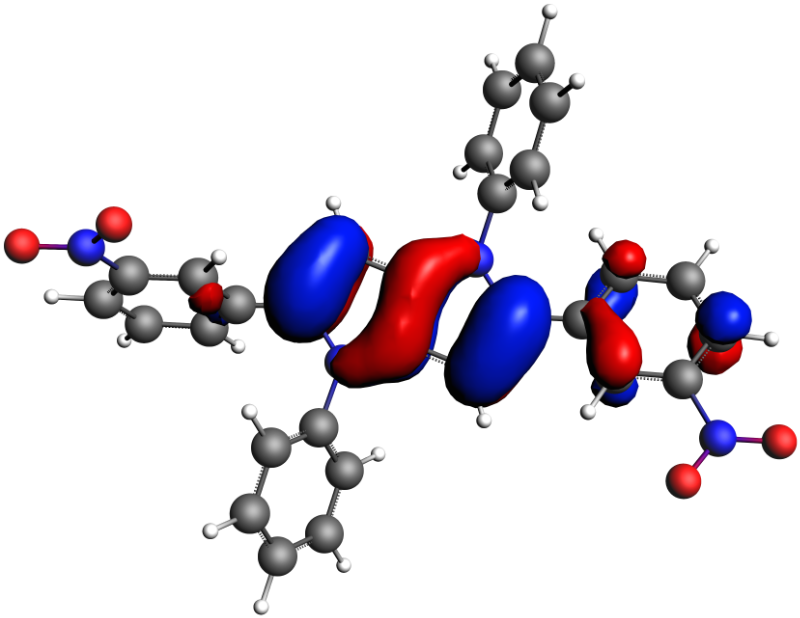 | 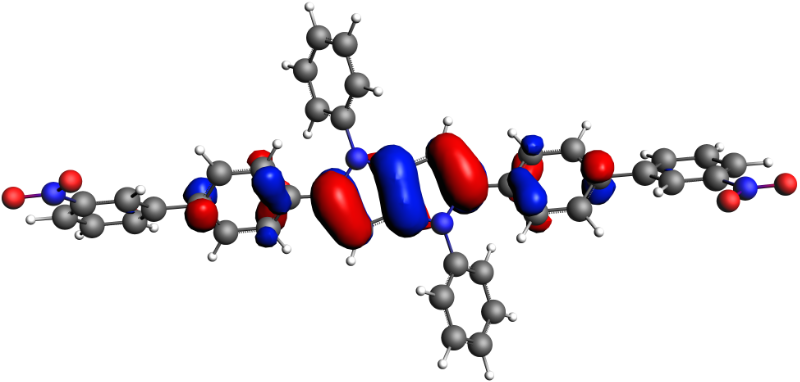 | 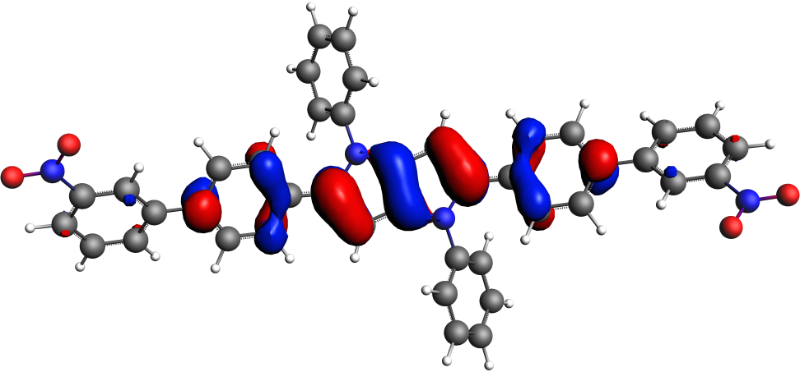 | | | | 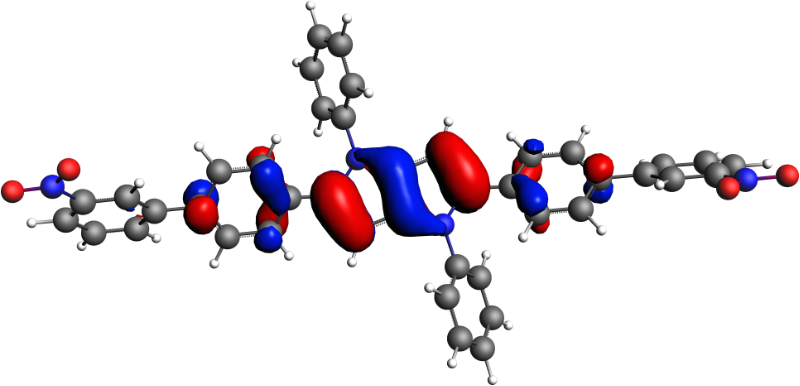 | | |
|  | **1o** | | | **2o** | | | | | | |
| **LUMO** | 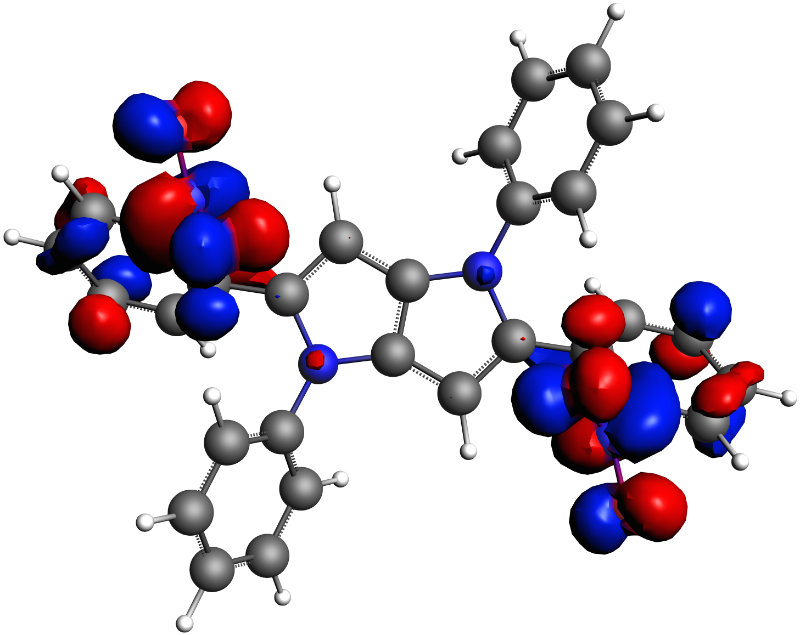 | 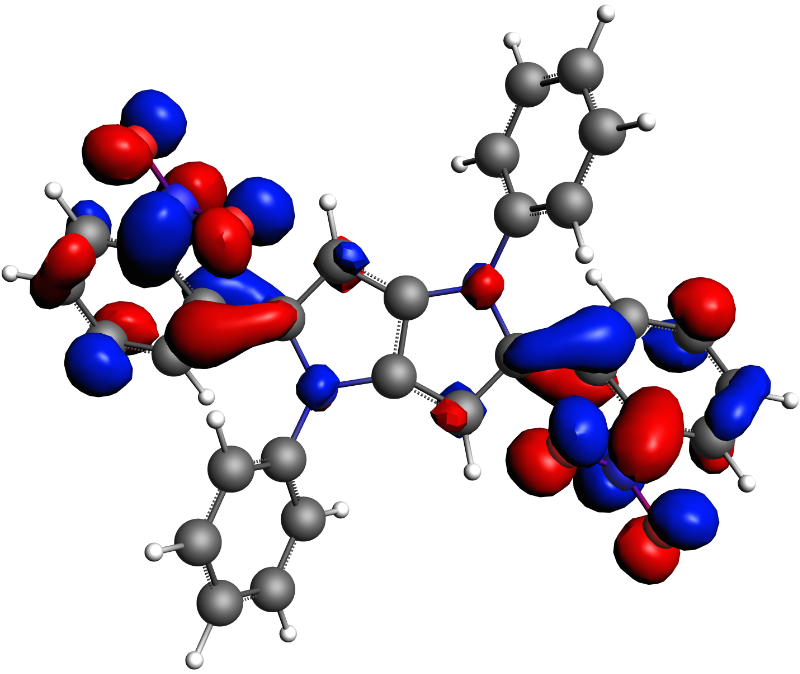 | 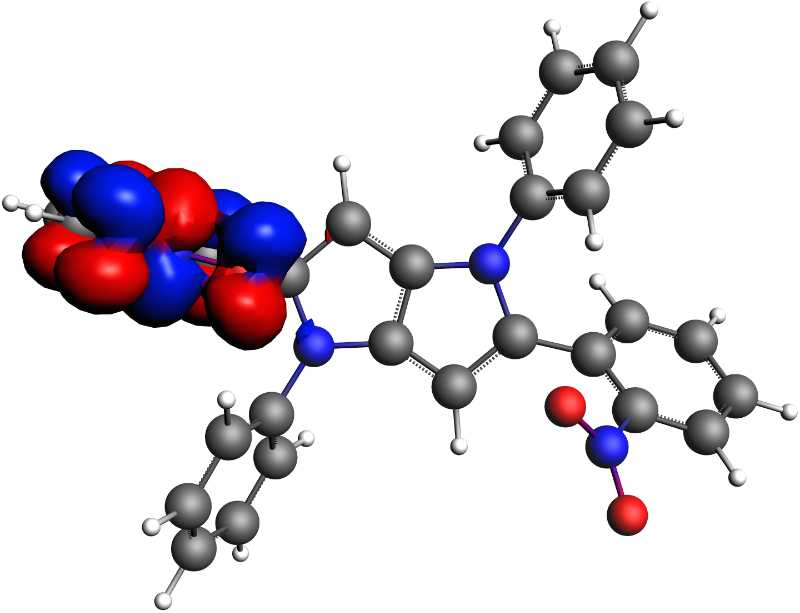 | 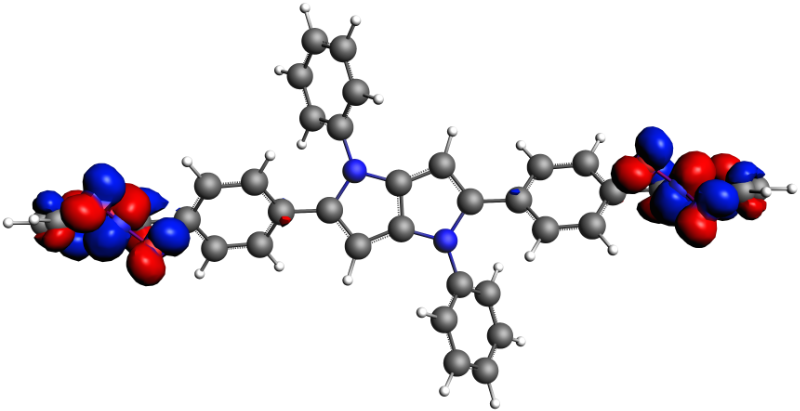 | | 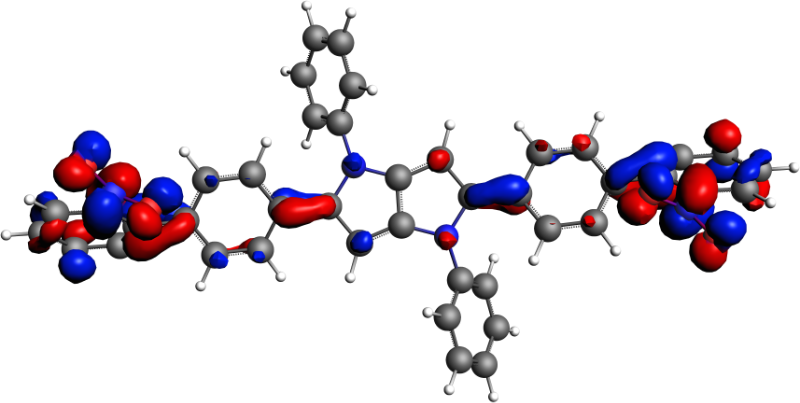 | | | | 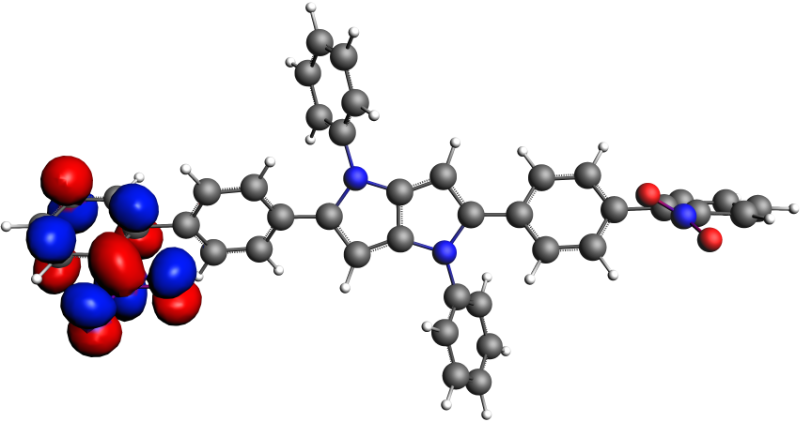 | |
| **HOMO** | 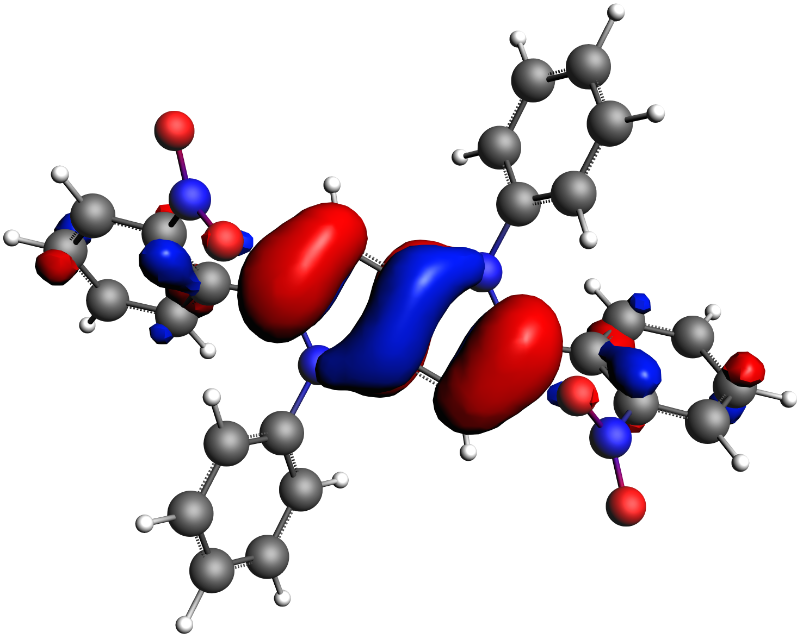 | 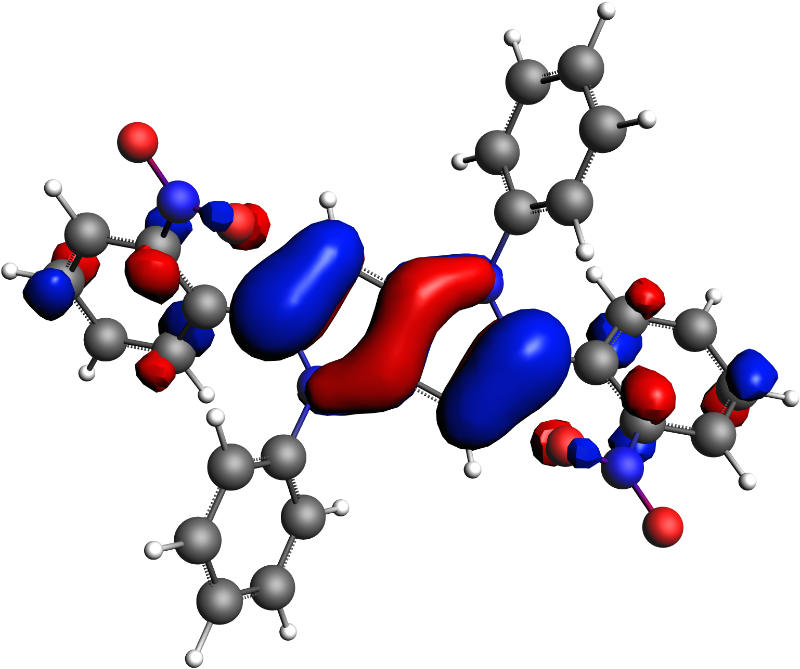 | 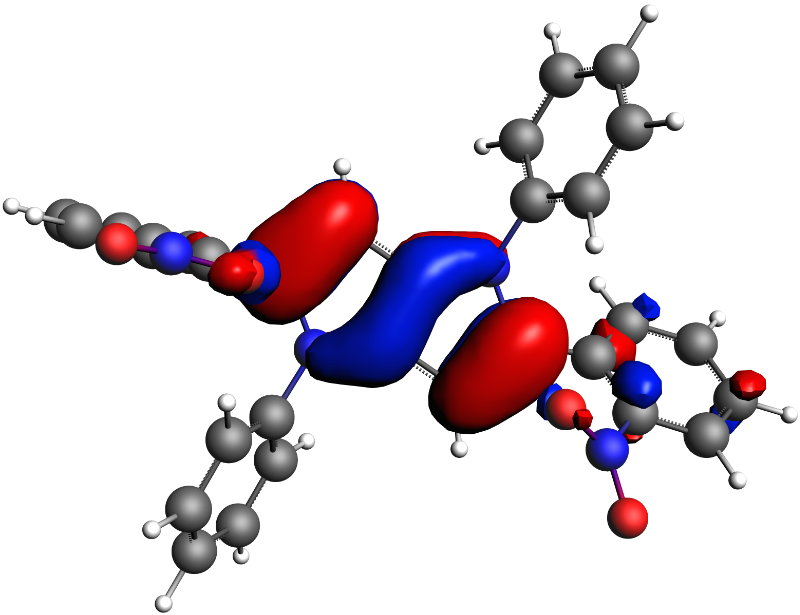 | 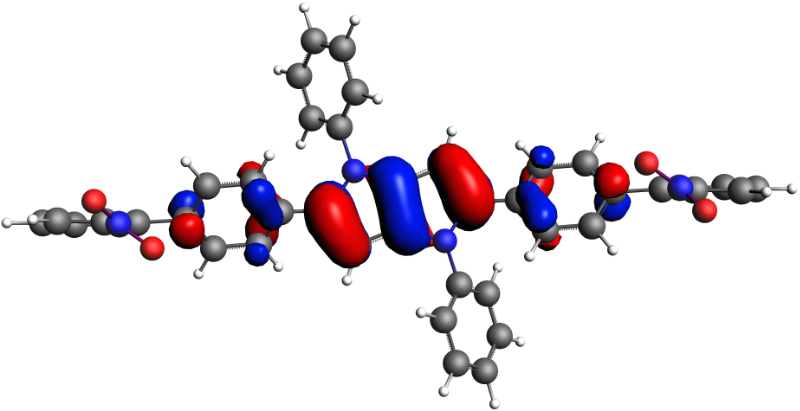 | | 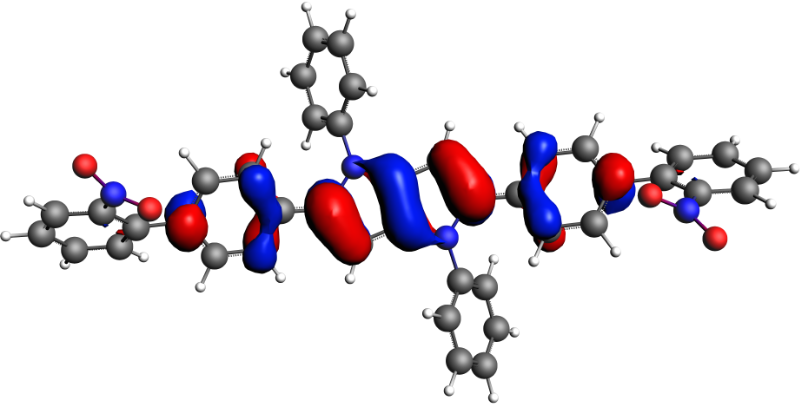 | | | | 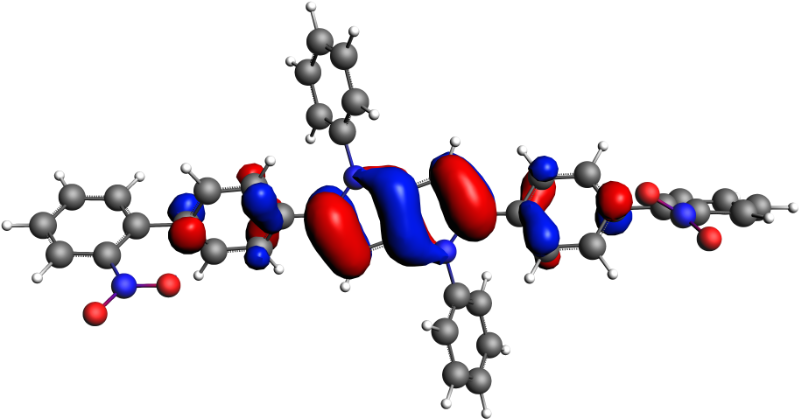 | |

**Supplementary Figure 10.** Selected molecular orbitals of TAPPs **1p**,**m**,**o** and **2p**,**m**,**o**. The alkyl substituents are replaced by H atoms for clarify.


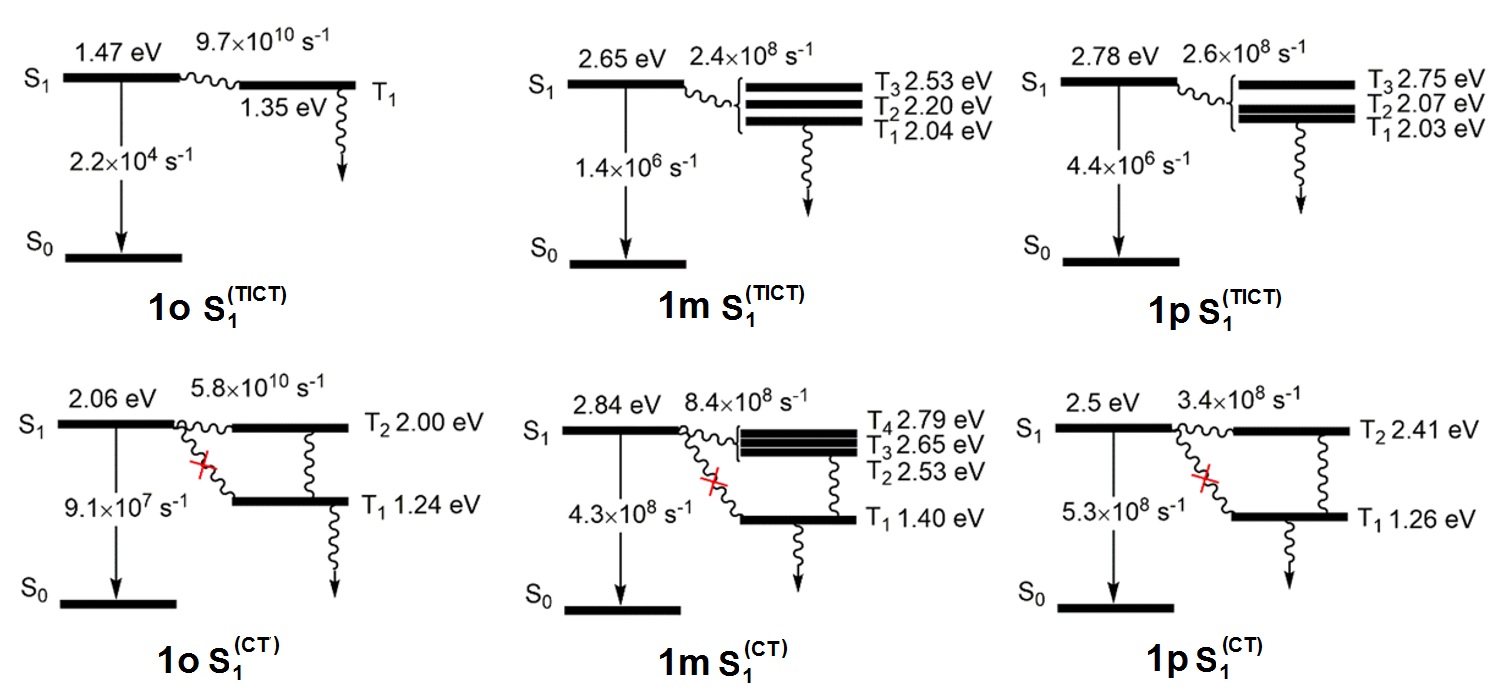


**Supplementary Figure 11.** The modified Jablonski diagram of TAPPs **1o**, **1m** and **1p**. The solid and wavy lines denote the radiative and radiationless transitions, respectively. The *k_r_* and $\sum_{j} k_{S_{1}\to T_{j}}$ rates are also presented in this Figure.


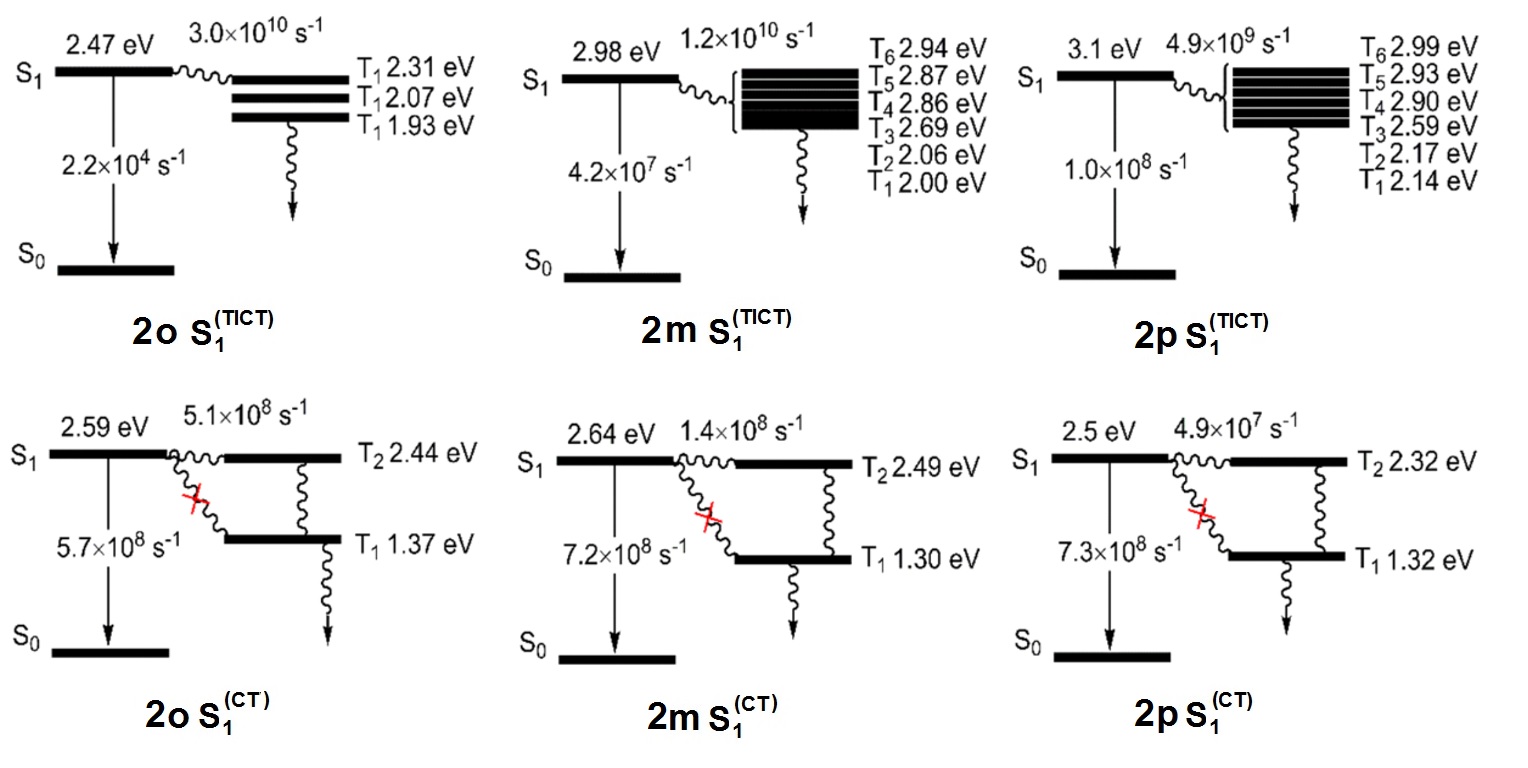


**Supplementary Figure 12.** The modified Jablonski diagram of TAPPs **2o**, **2m** and **2p**. The solid and wavy lines denote the radiative and radiationless transitions, respectively. The *k_r_* and $\sum_{j} k_{S_{1}\to T_{j}}$rates are also presented in this Figure.

**Supplementary Table 2.** The values of extinction coefficient calculated at the absorption maximum (*ε_theor_*) as compared with the experimental data (*ε_exp_*) for TAPPs **1o**,**m**,**p** and **2o**,**m**,**p**.

| TAPP | *ε_exp_* at first absorption maximum, M^-1^ cm^-1^ | *ε_theor_* at *E*(S_0_-S_1_), M^-1^ cm^-1^ |
| --- | --- | --- |
| **1o** | 6.2×10^3^ | 1.7×10^4^ |
| **1m** | 3.7×10^4^ | 2.1×10^4^ |
| **1p** | 4.7×10^4^ | 1.1×10^5^ |
| **2o** | 3.6×10^4^ | 9.6×10^4^ |
| **2m** | 4.7×10^4^ | 1.5×10^5^ |
| **2p** | 5.0×10^4^ | 1.6×10^5^ |

**Supplementary Table 3.** Computed dihedral angles between pyrrolo-pyrrole core and nitrophenyl groups for TAPPs **1o**,**m**,**p** and **2o**,**m**,**p**.

| TAPP | θ_1_, deg. | | | θ_2_, deg. | | | θ_3_, deg. | | | θ_4_, deg. | | |
| --- | --- | --- | --- | --- | --- | --- | --- | --- | --- | --- | --- | --- |
|  | S_0_ | S_1_CT | S_1_TICT | S_0_ | S_1_CT | S_1_TICT | S_0_ | S_1_CT | S_1_TICT | S_0_ | S_1_CT | S_1_TICT |
| **1o** | 54.5 | 42.7 | 54.7 | 54.5 | 42.7 | 95.4 | - | - | - | - | - | - |
| **1m** | 38.0 | 21.5 | 29.3 | 38.0 | 21.5 | 66.6 | - | - | - | - | - | - |
| **1p** | 36.0 | 20.9 | 29.9 | 36.0 | 20.9 | 87.5 | - | - | - | - | - | - |
| **2o** | 39.3 | 21.4 | 33.1 | 39.3 | 21.4 | 32.1 | 49.5 | 42.1 | 89.7 | 49.5 | 42.1 | 54.8 |
| **2m** | 38.3 | 20.5 | 29.7 | 38.3 | 20.6 | 38.0 | 35.6 | 28.1 | 36.0 | 35.6 | 28.2 | 39.3 |
| **2p** | 37.9 | 20.8 | 93.5 | 37.9 | 20.8 | 28.8 | 35.0 | 26.3 | 30.7 | 35.0 | 26.5 | 37.4 |

**Supplementary Table 4.** Parameters of ISC deactivation channels for TAPPs **1o**,**m**,**p** and **2o**,**m**,**p**.

| Molecule | *j* ^a^ | $\langle\text{S}_{1}\vert\hat{\text{H}}_{\text{SO}}\vert\text{T}_{\text{j}}\rangle$, cm-^1^ | Δ*E*(S_1_-T*_j_*), eV | $\text{k}_{\text{S}_{1}\to\text{T}_{\text{j}}}$, s^-1^ | Assignment/Weight^a^ (T*_j_*) |
| --- | --- | --- | --- | --- | --- |
| **1o** (S_1_^(TICT)^) | 1 | 5.48 | 0.12 | 9.7×10^10^ | HOMO-LUMO/0.855 |
| **1m** (S_1_^(TICT)^) | 1 | 3.57 | 0.61 | 5.7×10^7^ | HOMO-LUMO+2/0.612 |
|  | 2 | 1.02 | 0.45 | 5.7×10^7^ | HOMO-13-LUMO/0.829 |
|  | 3 | 0.20 | 0.12 | 1.3×10^8^ | HOMO-LUMO/0.777 |
| **1p** (S_1_^(TICT)^) | 1 | 1.45 | 0.75 | 3.6×10^6^ | HOMO-LUMO+1/0.637 |
|  | 2 | 6.93 | 0.71 | 1.1×10^8^ | HOMO-16-LUMO/0.848 |
|  | 3 | 0.16 | 0.03 | 1.5×10^8^ | HOMO-LUMO/0.821 |
| **1o** (S_1_^(CT)^) | 1 | 0.23 | 0.82 | 1.1×10^4^ | HOMO-LUMO/0.787 |
|  | 2 | 3.45 | 0.06 | 5.8×10^10^ | HOMO-LUMO+1/0.784 |
| **1m** (S_1_^(CT)^) | 1 | 0.00 | 1.44 | 0.00 | HOMO-LUMO/0.412  HOMO-LUMO+2/0.474 |
|  | 2 | 0.21 | 0.31 | 1.9×10^7^ | HOMO-LUMO+1/0.581 |
|  | 3 | 0.11 | 0.19 | 2.4×10^7^ | HOMO-LUMO/0.274  HOMO-LUMO+2/0.264 |
|  | 4 | 0.39 | 0.05 | 8.0×10^8^ | HOMO-13-LUMO/0.357  HOMO-14-LUMO+2/0.245 |
| **1p** (S_1_^(CT)^) | 1 | 0.01 | 1.24 | 2.5×10^2^ | HOMO-LUMO/0.806 |
|  | 2 | 0.3 | 0.09 | 3.6×10^8^ | HOMO-LUMO+1/0.594 |
| **2o** (S_1_^(TICT)^) | 1 | 29.7 | 0.54 | 2.2×10^10^ | HOMO-16-LUMO/0.850 |
|  | 2 | 2.43 | 0.40 | 1.0×10^9^ | HOMO-LUMO+2/0.693 |
|  | 3 | 1.63 | 0.16 | 6.5×10^9^ | HOMO-LUMO/0.476 |
| **2m** (S_1_^(TICT)^) | 1 | 2.47 | 0.98 | 1.3×10^5^ | HOMO-17-LUMO/0.841 |
|  | 2 | 0.28 | 0.92 | 4.9×10^3^ | HOMO-LUMO+2/0.694 |
|  | 3 | 0.32 | 0.29 | 6.8×10^7^ | HOMO-LUMO/0.319 |
|  | 4 | 0.63 | 0.12 | 1.4×10^9^ | HOMO-18-LUMO+1/0.641 |
|  | 5 | 1.64 | 0.11 | 1.0×10^10^ | HOMO-15-LUMO/0.779 |
|  | 6 | 0.26 | 0.04 | 4.2×10^8^ | HOMO-LUMO+3/0.207 |
| **2p** (S_1_^(TICT)^) | 1 | 1.10 | 0.68 | 1.4×10^4^ | HOMO-LUMO+2/0.489 |
|  | 2 | 1.04 | 0.1 | 5.3×10^2^ | HOMO-17-LUMO/0.792 |
|  | 3 | 0.41 | 0.38 | 5.7×10^6^ | HOMO-2-LUMO/0.571 |
|  | 4 | 0.24 | 0.35 | 2.1×10^8^ | HOMO-18-LUMO+1/0.722 |
|  | 5 | 0.23 | 1.28 | 4.0×10^9^ | HOMO-15-LUMO/0.810 |
|  | 6 | 0.16 | 0.41 | 6.5×10^8^ | HOMO-3-LUMO+1/0.280 |
| **2o** (S_1_^(CT)^) | 1 | 1.22 | 0.29 | 2.5×10^1^ | HOMO-LUMO+2/0.429  HOMO-LUMO/0.419 |
|  | 2 | 0.15 | 0.43 | 5.1×10^8^ | HOMO-LUMO+1/0.328  HOMO-2-LUMO/0.211 |
| **2m** (S_1_^(CT)^) | 1 | 1.34 | 0.03 | 2.8×10^-2^ | HOMO-LUMO+2/0.514  HOMO-LUMO/0.323 |
|  | 2 | 0.15 | 0.23 | 1.4×10^8^ | HOMO-LUMO+3/0.376  HOMO-2-LUMO/0.171 |
| **2p** (S_1_^(CT)^) | 1 | 1.18 | 0.02 | 3.0×10^-1^ | HOMO-LUMO+2/0.324  HOMO-LUMO/0.546 |
|  | 2 | 0.18 | 0.15 | 4.9×10^7^ | HOMO-LUMO+1/0.353  HOMO-2-LUMO/0.268 |

^a^ For all TAPPs the S_1_ state corresponds to the HOMO-LUMO excitation independently on the S_1_^(CT)^ or S_1_^(TICT)^. The weight of HOMO-LUMO configuration varies in the region 0.92-0.94 for molecules **1o**,**m**,**p** and in the region 0.5-0.95 for the molecules **2o**,**m**,**p**.

1. **Time correlated single photon counting experiments**

Time correlated single photon counting (TCSPC) experiments conducted with Horiba Jobin Yvon Fluorolog-3-22 spectrofluorometer. Excitation achieved via pulsed laser diode NanoLED at a repetition rate of 1MHz frequency where wavelength excitation *λ_ex_* = 406 nm and decays monitored at the emission maxima for each nitropyrrolopyrrole derivative.

a
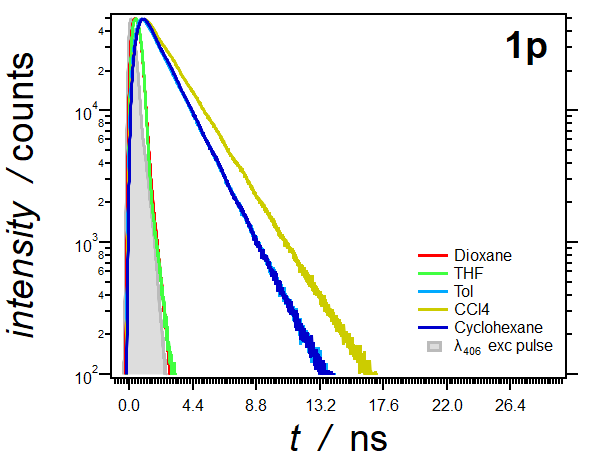
b
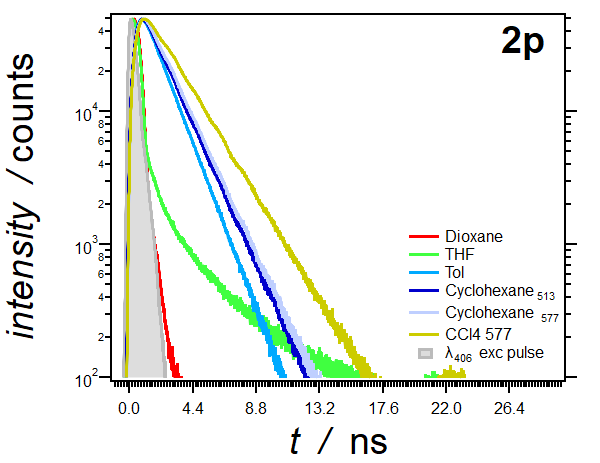


c
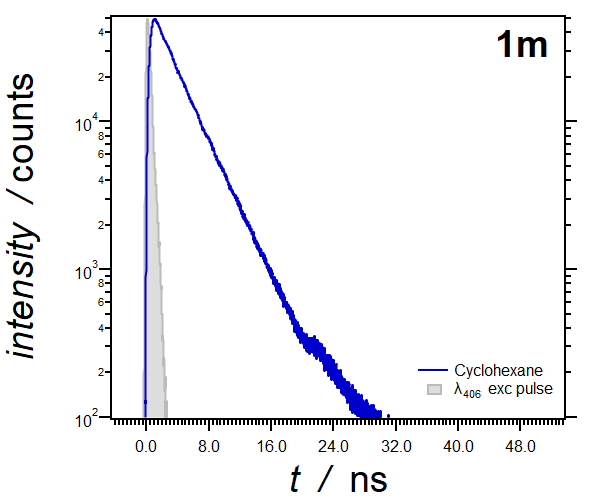
 d
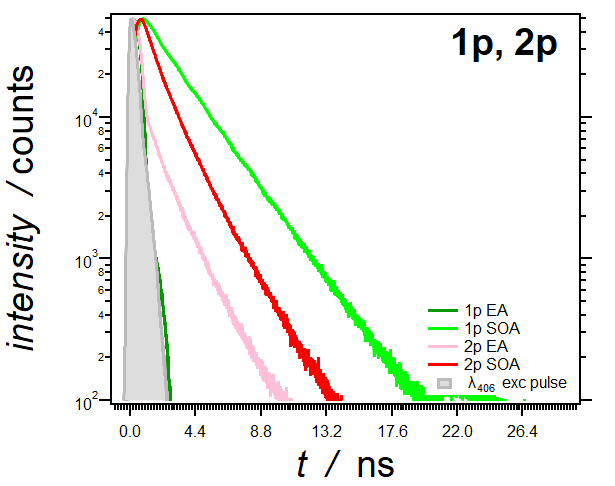


**Supplementary Figure 13.** Time correlated single photon counting of nitropyrrolopyrrole derivatives. Fluorescence decays fits resolved via deconvolution to obtain emission time constants used to calculate radiative and non-radiative decay rates.

1. **Transient-absorption spectroscopy**

A Helios pump-probe spectrometer (Ultrafast Systems, LLC, Florida, USA) is used for recording in transmission mode the transient absorption spectra. 800-nm pulses (>35 fs, 4.0 mJ per pulse, at 1 kHz) are generated by a SpitFire Pro 35F regenerative amplifier (Spectra Physics, Newport, CA, USA). The amplifier is pumped with an Empower 30 Q-switched laser ran at 20 W. A MaiTai SP oscillator provides the seed beam (55 nm bandwidth). The wavelength of the pump is tuned using an optical parametric amplifier, OPA-800CU (Newport Corporation, Newport, CA, USA), equipped with harmonic generators. Responses from pure solvents are used for the chirp correction of the transient-absorption data (Supplementary Figure 14).

**a**
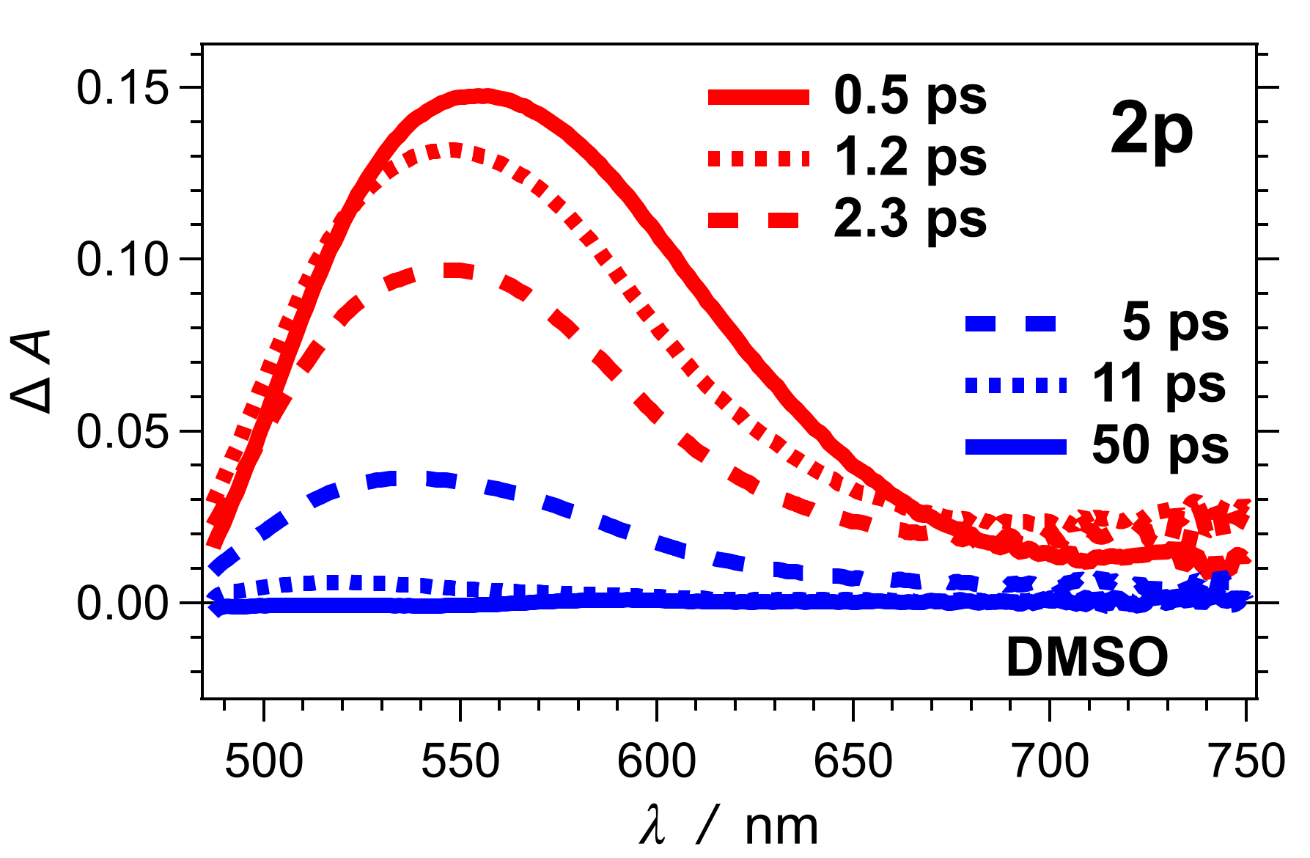
 **b**
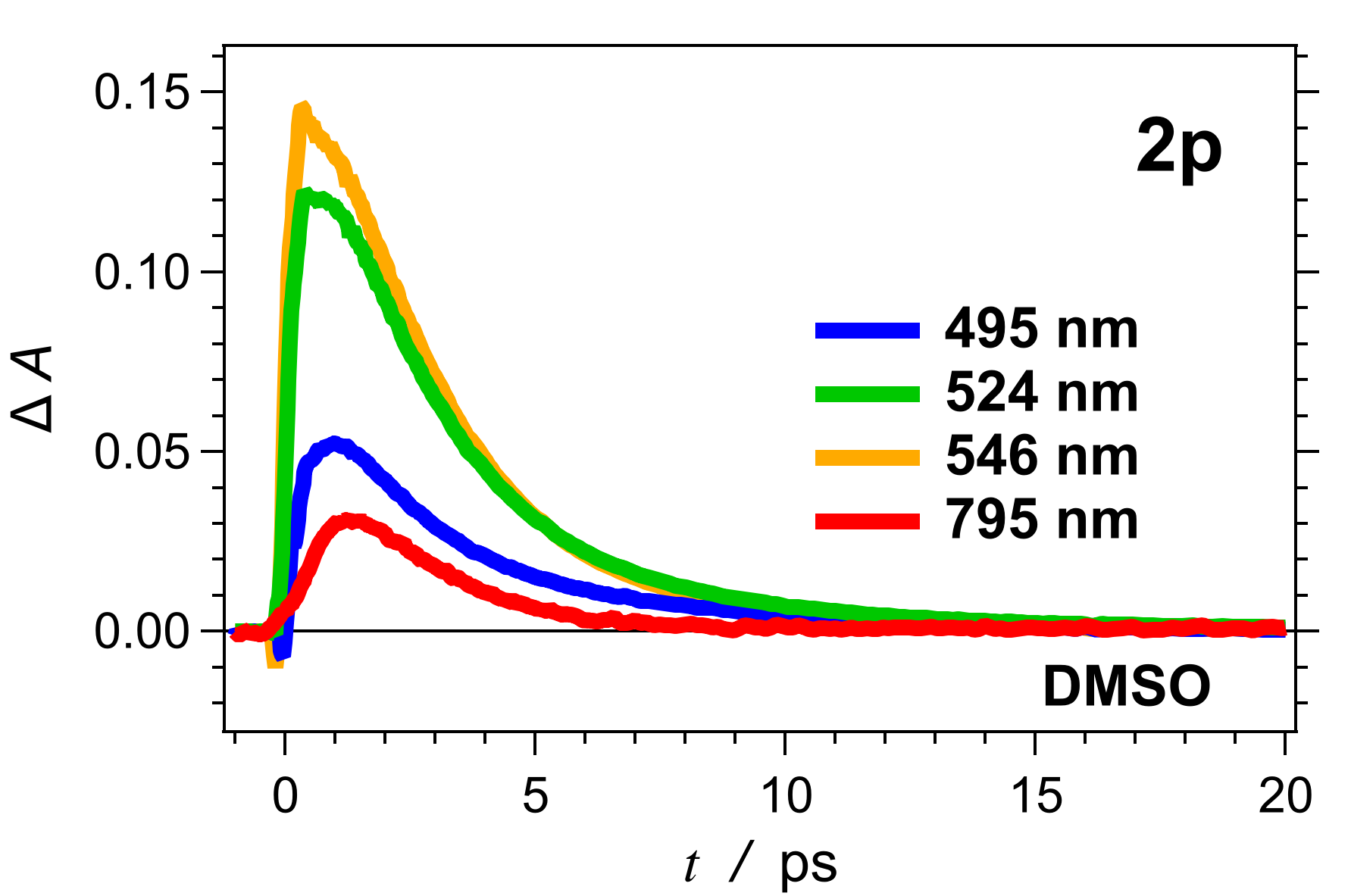


**Supplementary Figure 14.** Transient absorption (TA) dynamics of **2p** for DMSO: (a) TA spectra recorded at different times; and (b) TA kinetic curves following the evolution of Δ*A* at different wavelengths. (*λ*_ex_ = 400 nm; 4 μJ per pulse at a repetition rate of 1 kHz, and the pump blocked every other pulse; pulse FWHM = 50 fs; data recorded in a transmission mode through a 2-mm quartz cuvette, which increases the effective instrument response time to 210 fs with *t*_0_ = 68 fs after the chirp correction).

Global analysis, aided by single value decomposition, allows for fitting each of the TA data sets, Δ*A*(*λ*, *t*), to multiple exponents (Supplementary Figure 15), as implemented by Surface Xplorer (Ultrafast Systems, LLC, Florida, USA):

|  | $\text{Δ}\text{Α}\left( \text{λ}\text{,}\text{ t} \right)\text{ = }\text{α}_{\infty}\left( \text{λ} \right)\text{ + }\sum_{\text{i}\text{ = 1}}^{\text{n}} \text{α}_{\text{i}}\left( \text{λ} \right)\text{ }\text{e}^{\text{– }\frac{\text{t }{- \text{t}}_{0}}{\text{τ}_{\text{i}}}}$ | (S6) |
| --- | --- | --- |

To gain further insights in the TA we carry out follow-up global analyses, introducing parallel pathways, using IgorPro v. 8 (WaveMetrics, Inc., Lake Oswego, OR, USA). For example, for multiple transitions from an initially detected excited state, S_1_^(I)^, to the ground state, S_0_, involving two more excited states, X and Y, we ascribe the following rate constants:

| S_1_^(I)^ ⟶ S_0_ | *k*_10_ | (S7a) |
| --- | --- | --- |
| S_1_^(I)^ ⟶ X | *k*_11_ | (S7b) |
| S_1_^(I)^ ⟶ Y | *k*_12_ | (S7c) |
| X ⟶ S_0_ | *k*_20_ | (S7d) |
| X ⟶ Y | *k*_21_ | (S7e) |
| Y ⟶ S_0_ | *k*_30_ | (S7f) |

Therefore, Δ*A*(*λ*, *t*) for each transient is expressed as:

|  | $\text{Δ}\text{Α}_{\text{S}_{1}^{\text{(I)}}}\left( \text{λ}\text{,}\text{ t} \right)\text{ = }\text{α}_{1}\left( \text{λ} \right)\text{ }\text{e}^{\text{– (}\text{k}_{10}\text{ + }\text{k}_{11}\text{ }\text{+ }\text{k}_{12}\text{)(}\text{t }{- \text{t}}_{0})}$ | (S8a) |
| --- | --- | --- |
|  | $\text{Δ}\text{Α}_{\text{X}}\left( \text{λ}\text{,}\text{ t} \right)\text{ = }\text{α}_{3}\left( \text{λ} \right)\text{ }\text{e}^{\text{– (}\text{k}_{20}\text{ + }\text{k}_{21}\text{)(}\text{t }{- \text{t}}_{0})}- \text{α}_{2}\left( \text{λ} \right)\text{ }\text{e}^{\text{– }\text{k}_{11}\text{(}\text{t }{- \text{t}}_{0})}$ | (S8b) |
|  | $\text{Δ}\text{Α}_{\text{Y}}\left( \text{λ}\text{,}\text{ t} \right)\text{ = }\text{α}_{5}\left( \text{λ} \right)\text{ }\text{e}^{\text{– }\text{k}_{30}\text{(}\text{t }{- \text{t}}_{0})}- \text{α}_{4}\left( \text{λ} \right)\text{ }\text{e}^{\text{– (}\text{k}_{12}\text{ + }\text{k}_{21}\text{)(}\text{t }{- \text{t}}_{0})}$ | (S8c) |
|  | $\text{Δ}\text{Α}_{\text{S}_{0}}\left( \text{λ}\text{,}\text{ t} \right)\text{ =}-\text{α}_{6}\left( \text{λ} \right)\text{ }\text{e}^{\text{– (}\text{k}_{10}\text{ + }\text{k}_{20}\text{ }\text{+ }\text{k}_{30}\text{)(}\text{t }{- \text{t}}_{0})}$ | (S8d) |

which allows for representing the total TA dynamics as:

| $\text{Δ}\text{Α}\left( \text{λ}\text{,}\text{ t} \right)\text{ = }\text{α}_{\infty}\left( \text{λ} \right)\text{ +}\text{ }\text{α}_{\text{S}_{1}^{\text{(I)}}}\text{ Δ}\text{Α}_{\text{S}_{1}^{\text{(I)}}}\left( \text{λ}\text{,}\text{ t} \right)+ \text{α}_{\text{X}}\text{ }\text{Δ}\text{Α}_{\text{X}}\left( \text{λ}\text{,}\text{ t} \right)+\text{α}_{\text{Υ}}\text{ }\text{Δ}\text{Α}_{\text{Υ}}\left( \text{λ}\text{,}\text{ t} \right)+\text{α}_{\text{S}_{0}} \text{Δ}\text{Α}_{\text{S}_{0}}\left( \text{λ}\text{,}\text{ t} \right)$ | (S8e) |
| --- | --- |

For the spectral range at wavelength longer than about 500 nm, $\text{α}_{\text{S}_{0}}$ has negligible contributions. For most data fits, $\text{α}_{\infty}\left( \text{λ} \right)$ converges to values close to zero. When $\text{α}_{\infty}\left( \text{λ} \right)$ ≠ 0, as for **1p** in cyclohexane, for example, we ascribe it to the TA of a long-lived transient, such as a triplet state. Introducing separate triplet-formation pathways to the data fits for cases like **1p** in cyclohexane, i.e., Δ*A*_T_(*λ*, *t*) = *α_j_*(λ)(1 – exp(– Σ*_i_ k_i_*_,ISC_(*t* – *t*_0_))) where the decay rate constants to ground state, *k_i_*_0_, are replaced by (*k_i_*_0_ + *k_i_*_,ISC_), does not lead to reliable convergences of *k_i_*_,ISC_. The values of the ISC rate constants for these cases are too small and outside the dynamic range of the used pump-probe technique.

When a component, *k_ij_*, of the composite rate constants is relatively small, i.e., *k_ij_* << Σ*_q_* *k_iq_*, it suggests for negligible to no contribution of that branch of the pathway to the TA dynamics, and we hold *k_ij_* at zero value. For example, *k*_10_ for each case shows negligible contributions to the kinetics, which indicates that IC or ISC of an initially formed singlet-excited state are much slower than its transformations along the S_1_ PES. As a result, *α*_1_(*λ*) ≈ *α*_2_(*λ*) (Supplementary Equations S8a and S8b), which equivalate to the *α*_1_(*λ*) values obtained from implementing Supplementary Equation S6 (Supplementary Figure 15, Table 2).

**a**
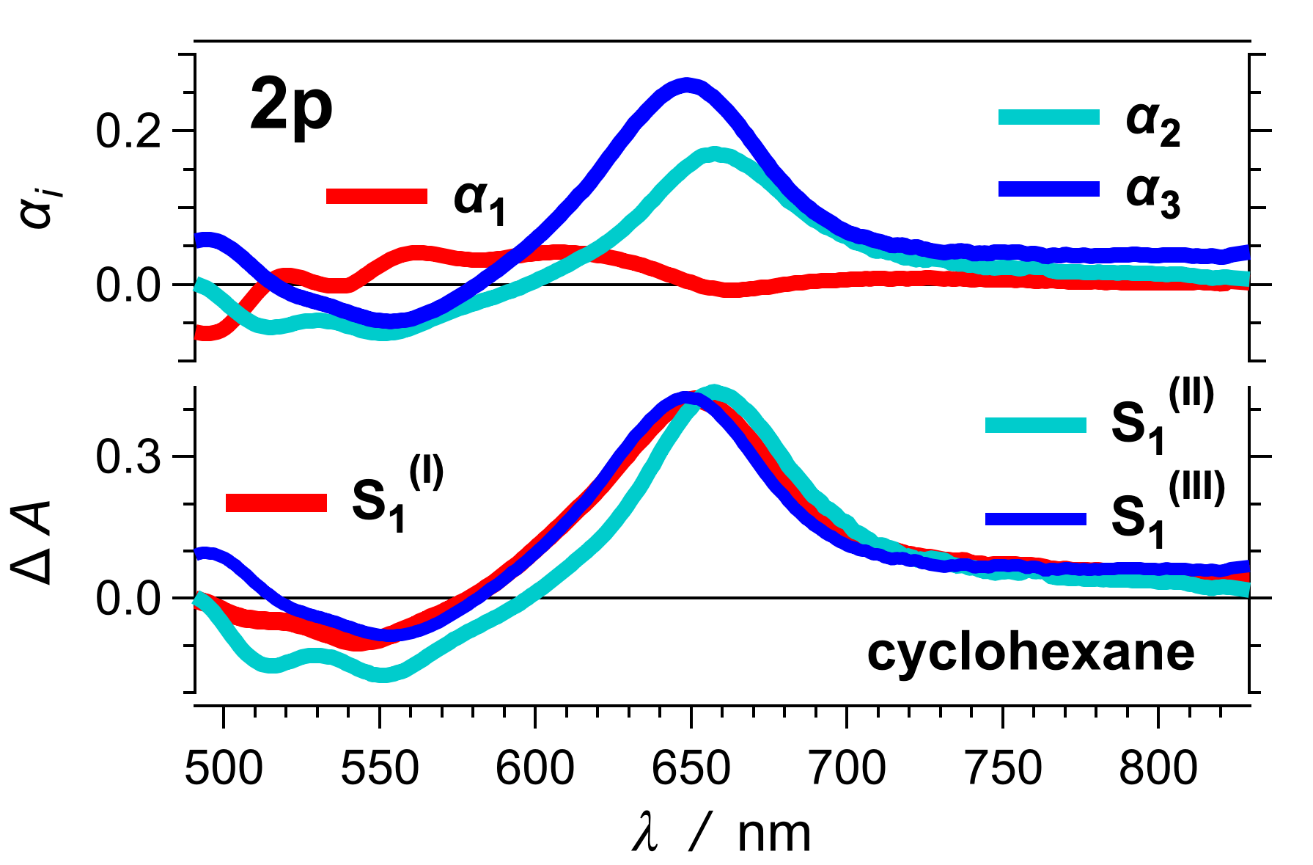


**b**
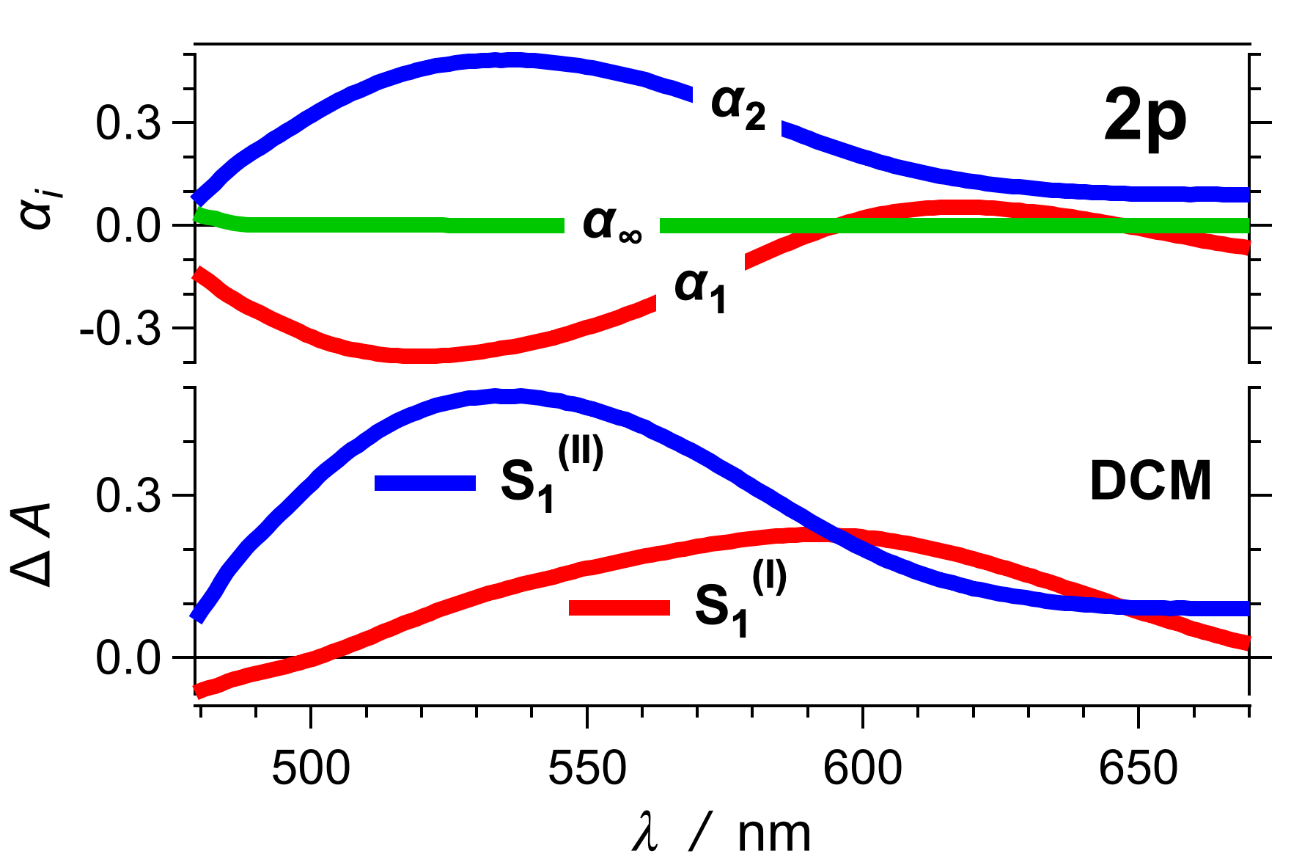
 **c**
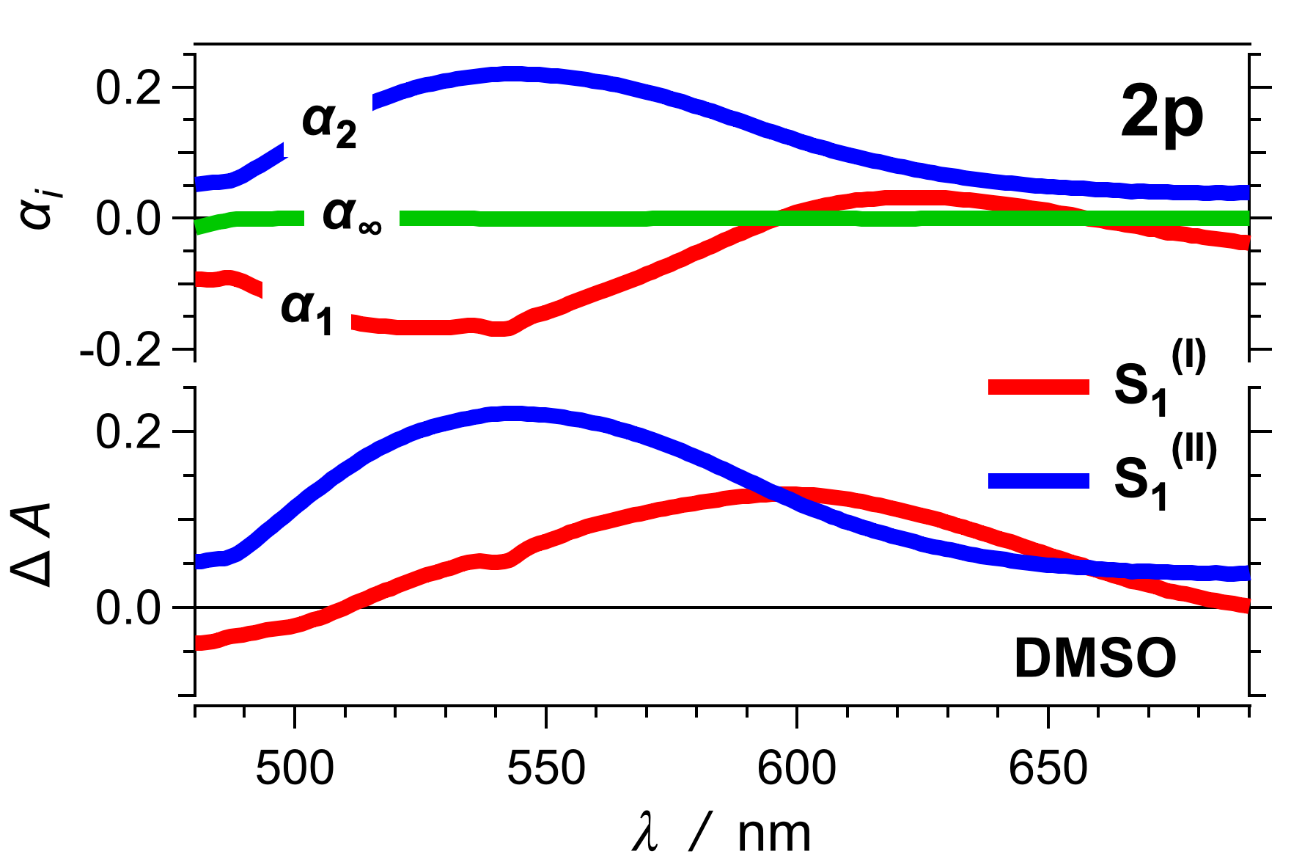


**Supplementary Figure 15.** Global-fit analysis of transient absorption (TA) spectra and kinetics of **2p** for (a) non-polar, and (b,c) polar solvents, obtained from implementation of Supplementary Equations S6 and S7. (a) All singlet-excited states of **2p** in cyclohexane have quite similar TA spectra. The convergence of the global fits suggests for *k*_10_ ≈ *k*_21_ ≈ 0 (Supplementary Equation S2). Therefore, *τ*_1_ = (*k*_11_ + *k*_12_)^–1^ = 1.8 ps with preexponential factors *α*_1_(*λ*); *τ*_2_ = *k*_20_^–1^ = 220 ps with preexponential factors *α*_2_(*λ*); and *τ*_3_ = *k*_30_^–1^ = 1.9 ns with preexponential factors *α*_3_(*λ*). Unrestricted global fits lead to convergence *τ*_3_ to a broad range a values ranging between about 1 and 3 ns. Therefore, we hold *τ*_3_ = 1.9 ns, which was obtained from deconvolution fits of the TCSPC emission decays of **1p** in cyclohexane (Table 1). (b,c) For **2p** in DCM and DMSO, the TA dynamics was successfully fit to a model with two singlet-excited states, i.e., *α*_Y_(*λ*) = 0 (Supplementary Equation S7e), and the convergence of the global fits suggests for *k*_10_ ≈ *k*_12_ ≈ 0 (Supplementary Equation S7), revealing that sequential transitions between the excited states dominate the TA dynamics. (b) *τ*_1_ = *k*_11_^–1^ = 0.68 ps with preexponential factors *α*_1_(*λ*); and *τ*_2_ = *k*_20_^–1^ = 2.4 ps with preexponential factors *α*_2_(*λ*). (b) *τ*_1_ = *k*_11_^–1^ = 0.41 ps with preexponential factors *α*_1_(*λ*); and *τ*_2_ = *k*_20_^–1^ = 2.7 ps with preexponential factors *α*_2_(*λ*).

**Supplementary References**

1 Janiga, A., Krzeszewski, M. & Gryko, D. T. Strongly Fluorescent Heterocycles And A Method For Their Synthesis. WO2014/70029 (2014).

2 Friese, D. H. *et al.* Pyrrolo[3,2-*b*]pyrroles—from unprecedented solvatofluorochromism to two-photon absorption. *Chem. Eur. J.* **21**, 18364-18374, (2015).

3 Rurack, K. in *Springer Series on Fluorescence* Vol. 5 (ed Wolfbeis, O. S.) 101–145 (Springer-Verlag, 2008).

4 Wurth, C., Grabolle, M., Pauli, J., Spieles, M. & Resch-Genger, U. Relative and absolute determination of fluorescence quantum yields of transparent samples. *Nat. Protoc.* **8**, 1535-1550, (2013).

5 Becke, A. D. Density-functional thermochemistry. III. The role of exact exchange. *J. Chem. Phys.* **98**, 5648-5652, (1993).

6 Lee, C., Yang, W. & Parr, R. G. Development of the Colle-Salvetti correlation-energy formula into a functional of the electron density. *Physical Review B* **37**, 785-789, (1988).

7 Ditchfield, R., Hehre, W. J. & Pople, J. A. Self‐Consistent Molecular‐Orbital Methods. IX. An Extended Gaussian‐Type Basis for Molecular‐Orbital Studies of Organic Molecules. *J. Chem. Phys.* **54**, 724-728, (1971).

8 Frisch, M. J., Pople, J. A. & Binkley, J. S. Self-consistent molecular orbital methods 25. Supplementary functions for Gaussian basis sets. *J. Chem. Phys.* **80**, 3265-3269, (1984).

9 Clark, T., Chandrasekhar, J., Spitznagel, G. W. & Schleyer, P. V. R. Efficient diffuse function‐augmented basis sets for anion calculations. III. The 3‐21+G basis set for first‐row elements, Li–F. *Journal of Computational Chemistry* **4**, 294-301, (1983).

10 Tomasi, J., Mennucci, B. & Cammi, R. Quantum Mechanical Continuum Solvation Models. *Chem. Rev.* **105**, 2999-3094, (2005).

11 Yanai, T., Tew, D. P. & Handy, N. C. A new hybrid exchange-correlation functional using the Coulomb-attenuating method (CAM-B3LYP). *Chem. Phys. Lett.* **393**, 51-57, (2004).

12 Wang, F. & Ziegler, T. A simplified relativistic time-dependent density-functional theory formalism for the calculations of excitation energies including spin-orbit coupling effect. *J. Chem. Phys.* **123**, 154102, (2005).

13 van Lenthe, E. & Baerends, E. J. Optimized Slater-type basis sets for the elements 1-118. *J Comput Chem* **24**, 1142-1156, (2003).

14 Pye, C. C. & Ziegler, T. An implementation of the conductor-like screening model of solvation within the Amsterdam density functional package. *Theor. Chim. Acta* **101**, 396-408, (1999).

15 Samanta, P. K., Kim, D., Coropceanu, V. & Brdas, J. L. Up-Conversion Intersystem Crossing Rates in Organic Emitters for Thermally Activated Delayed Fluorescence: Impact of the Nature of Singlet vs Triplet Excited States. *J. Am. Chem. Soc.* **139**, 4042-4051, (2017).

16 van Lenthe, E., Snijders, J. G. & Baerends, E. J. The zero‐order regular approximation for relativistic effects: The effect of spin–orbit coupling in closed shell molecules. *J. Chem. Phys.* **105**, 6505-6516, (1996).

17 van Lenthe, E., van Leeuwen, R., Baerends, E. J. & Snijders, J. G. Relativistic regular two-component Hamiltonians. *Int. J. Quantum Chem.* **57**, 281-293, (1996).

18 Mori, K. & Goumans, T. P. M. a. Predicting phosphorescent lifetimes and zero-field splitting of organometallic complexes with time-dependent density functional theory including spin-orbit coupling. *Phys. Chem. Chem. Phys.* **16**, 14523-14530, (2014).

19 Baryshnikov, G., Minaev, B. & Agren, H. Theory and calculation of the phosphorescence phenomenon. *Chem. Rev.* **117**, 6500-6537, (2017).

20 Plotnikov, V. G. Regularities of the processes of radiationless conversion in polyatomic molecules. *Int. J. Quantum Chem.* **16**, 527-541, (1979).

21 Baryshnikov, G. V. *et al.* Benzoannelated aza-, oxa- and azaoxa 8 circulenes as promising blue organic emitters. *Phys. Chem. Chem. Phys.* **18**, 28040-28051, (2016).

22 Valiev, R. R., Cherepanov, V. N., Artyukhov, V. Y. & Sundholm, D. Computational studies of photophysical properties of porphin, tetraphenylporphyrin and tetrabenzoporphyrin. *Phys. Chem. Chem. Phys.* **14**, 11508-11517, (2012).

23 Artyukhov, V. Y. *et al.* A combined theoretical and experimental study on molecular photonics. *Russian Physics Journal* **51**, 1097-1111, (2008).

24 Valiev, R. R. *et al.* The computational and experimental investigations of photophysical and spectroscopic properties of BF2 dipyrromethene complexes. *Spectrochimica Acta - Part A: Molecular and Biomolecular Spectroscopy* **117**, 323-329, (2014).

25 Valiev, R. R., Cherepanov, V. N., Baryshnikov, G. V. & Sundholm, D. First-principles method for calculating the rate constants of internal-conversion and intersystem-crossing transitions. *Phys. Chem. Chem. Phys.* **20**, 6121-6133, (2018).

26 Valiev, R. R. Competition between the nonadiabatic electronic state-mixing and the Herzberg-Teller vibronic effects in fluorescence process of tetraoxa[8]circulene. *Chem. Phys. Lett.* **738**, 136914, (2020).

27 Valiev, R. R., Cherepanov, V. N., Nasibullin, R. T., Sundholm, D. & Kurten, T. Calculating rate constants for intersystem crossing and internal conversion in the Franck-Condon and Herzberg-Teller approximations. *Phys. Chem. Chem. Phys.* **21**, 18495-18500, (2019).

28 ADF2018, SCM, Theoretical Chemistry (Vrije Universiteit, Amsterdam, The Netherlands, 2018).

29 Gaussian 16 Revision C.01 (Gaussian, Inc., Wallingford CT, 2016).
