# Supplementary material for: Deciphering the unusual fluorescence in weakly coupled bis-nitro-pyrrolo[3,2-b]pyrroles
Source: Commun Chem. 2020 Dec 17;3:190. doi: 10.1038/s42004-020-00434-6 (PMC9814504; doi:10.1038/s42004-020-00434-6)
Supplement: Supplementary file 2 — Description of Additional Supplementary Files [file 42004_2020_434_MOESM2_ESM.pdf]

Description of Additional Supplementary Files

File Name: Supplementary Data 1
Description: Detailed info on absorption spectrum of TAPP **1o** calculated by TDDFT/B3LYP/6-31+G(d) method with accounting of solvent effect (hexane)

File Name: Supplementary Data 2
Description: Detailed info on absorption spectrum of TAPP **1m** calculated by TDDFT/B3LYP/6-31+G(d) method with accounting of solvent effect (hexane)

File Name: Supplementary Data 3
Description: Detailed info on absorption spectrum of TAPP **1p** calculated by TDDFT/B3LYP/6-31+G(d) method with accounting of solvent effect (hexane)

File Name: Supplementary Data 4
Description: Detailed info on absorption spectrum of TAPP **2o** calculated by TDDFT/B3LYP/6-31+G(d) method with accounting of solvent effect (hexane)

File Name: Supplementary Data 5
Description: Detailed info on absorption spectrum of TAPP **2m** calculated by TDDFT/B3LYP/6-31+G(d) method with accounting of solvent effect (hexane)

File Name: Supplementary Data 6
Description: Detailed info on absorption spectrum of TAPP **2p** calculated by TDDFT/B3LYP/6-31+G(d) method with accounting of solvent effect (hexane)
